# An analysis of security vulnerabilities in container images for scientific data analysis

**DOI:** 10.1093/gigascience/giab025

**Published:** 2021-06-03

**Authors:** Bhupinder Kaur, Mathieu Dugré, Aiman Hanna, Tristan Glatard

**Affiliations:** Department of Computer Science and Software Engineering, Concordia University, Montreal, QC H3G 1M8, Canada; Department of Computer Science and Software Engineering, Concordia University, Montreal, QC H3G 1M8, Canada; Department of Computer Science and Software Engineering, Concordia University, Montreal, QC H3G 1M8, Canada; Department of Computer Science and Software Engineering, Concordia University, Montreal, QC H3G 1M8, Canada

**Keywords:** containers, Docker, singularity, security vulnerabilities, neuroimaging

## Abstract

**Background:**

Software containers greatly facilitate the deployment and reproducibility of scientific data analyses in various platforms. However, container images often contain outdated or unnecessary software packages, which increases the number of security vulnerabilities in the images, widens the attack surface in the container host, and creates substantial security risks for computing infrastructures at large. This article presents a vulnerability analysis of container images for scientific data analysis. We compare results obtained with 4 vulnerability scanners, focusing on the use case of neuroscience data analysis, and quantifying the effect of image update and minification on the number of vulnerabilities.

**Results:**

We find that container images used for neuroscience data analysis contain hundreds of vulnerabilities, that software updates remove roughly two-thirds of these vulnerabilities, and that removing unused packages is also effective.

**Conclusions:**

We provide recommendations on how to build container images with fewer vulnerabilities.

## Introduction

Software containers have emerged as an efficient solution to deploy scientific data analyses on various platforms, owing to their portability, ease of use, and limited overhead. Taking advantage of core Linux kernel features such as namespaces, control groups, and chroot, containers isolate processes from the host computer and often can control the memory, CPU, network, and file system resources assigned to them. However, containers still share the kernel, mounted file systems, and some devices with the host, which raises security concerns [1–3] and opens the door to privilege escalation, denial of service, information leak, and other types of attacks [4].

Container images typically include full operating system (OS) distributions in addition to data analysis software and their dependencies. They are rarely updated owing to concerns that updated software may be incompatible or may interfere with the results via numerical perturbations propagating in the analyses in unknown ways [5,6]. Images also typically include more dependencies than required, to make them easier to reuse between experiments. As a result, >30% of official images in DockerHub have been shown to contain high-priority security vulnerabilities [7], images on average contain >180 vulnerabilities [8], and vulnerabilities are often caused by outdated packages [9].

Scientific data analyses typically involve a range of computational infrastructures, including personal workstations, laboratory servers, high-performance computing clusters, and cloud computing platforms. It is common for researchers to have access to multiple systems through a combination of credentials and to migrate analyses depending on their evolution. As a result, an attacker gaining access to 1 of these systems, possibly through a vulnerable container, might be able to compromise an extensive infrastructure and use it for malicious purposes.

In this study, we focus on the vulnerabilities present in container images used in scientific data analysis, in particular in the neuroimaging domain. We address the following questions:

(i) What is the current amount of vulnerabilities in container images used in scientific analyses? Vulnerabilities are possible attack vectors that can seriously compromise the security of computing systems and the integrity of user data. We report vulnerability scans produced by 4 popular image scanning tools: Anchore, Vuls, Clair, and Singularity.

(ii) Can the amount of vulnerabilities be reduced by updating the images? To avoid breaking software dependencies and introducing numerical perturbations, container images used in scientific analyses often include outdated software. We report on the effect of software updates on the amount of vulnerabilities found in images.

(iii) Can the amount of vulnerabilities be reduced by minifying images? Container images often include more software packages than necessary for a typical analysis. We report on the impact of unused software packages on the presence of vulnerabilities.

The remainder of this article describes the container images and scanners used in our experiment, and our methodology for updating and minifying images. The results section presents the vulnerabilities detected in container images, quantifies the effectiveness of updating and minifying images, and explains the differences observed between scanners. In conclusion, we provide a set of image creation guidelines for a more secure deployment of containers in scientific analyses.

## Materials and Tools

We used container images from 2 popular application frameworks, as well as 4 of the major image scanners.

### Container images

We scanned all container images available at the time of this study on 2 containerization frameworks used in neuroscience: BIDS apps [10] (26 images) and Boutiques [11] (18 images), totaling 44 container images. At the time of the study, BIDS apps had 27 images, of which 1 was not available on DockerHub. Boutiques had 49 images; however, only 23 unique images were listed, of which 3 could not be retrieved and 2 were already included in BIDS apps. All the final 26 images from BIDS apps were Docker images, whereas the 18 Boutiques images contained 12 Docker images and 6 Singularity images.

### Image scanners

We compared the results obtained with 4 container image scanners: Anchore, Vuls, and Clair to scan Docker images and Singularity Container Tools (Stools) to scan Singularity images.

Anchore [12] is an end-to-end, open-source container security platform. It analyzes container images and lists vulnerable OS packages, non-OS packages (Python, Java, Gem, and npm), and files. In our experiments, we used Anchore Engine version 0.5.0 through Docker image anchore/anchore-engine:v0.5.0 and Anchore vulnerability database version 0.0.11.

Vuls [13]is an open-source vulnerability scanner for Linux and FreeBSD. It offers both static and dynamic scanning and both local and remote scanning. In our experiments, we used Vuls 0.9.0, executed through Docker image vuls/vuls:0.9.0 in remote dynamic mode.

Clair [14] an open-source and extensible vulnerability scanner for Docker and OCI (Open Container Initiative) container images, developed by CoreOS (now Container Linux), a Linux distribution to deploy container clusters. We used Clair through Clair-scanner [15] a tool to facilitate the testing of container images against a local Clair server. We used Clair version 2.0.6, executed through Docker image arminc/clair-local-scan:v2.0.6. For the vulnerability database, we used Docker image arminc/clair-db:latest, last updated on 18 September 2019.

Singularity Tools (Stools) [16] is an extension of Clair for Singularity images. Stools exports Singularity images to tar.gz format, acting as a single-layer Docker image to circumvent the Docker-specific requirements in the Clair API. In our experiments, we used Singularity Tools version 3.2.1 through Docker image vanessa/stools-clair:v3.2.1. Because Stools uses Clair internally for scanning, the vulnerability databases used by Stools are the same as mentioned for Clair. To scan Singularity images, we followed the steps mentioned in the Stools documentation [17].

### Vulnerability databases

Scanners refer to 2 types of vulnerability databases (Table 1). The first is the Open Vulnerability and Assessment Language (OVAL) database, an international open standard that supports various OS distributions including Ubuntu, Debian, and CentOS but not Alpine. The second is vulnerability databases from specific OS distributions, such as Alpine-SecDB, Debian Security Bug Tracker, Ubuntu CVE Tracker, or Red Hat Security Data. In these databases, OS distributions often assign a status to each vulnerability to keep track of required and available security fixes in different versions of the distribution. Vuls uses OVAL databases for all distributions except Alpine. On the contrary, Clair exclusively refers to distribution-specific databases. Anchore uses OVAL only for CentOS because distribution-specific databases are assumed to be more complete. It is also worth noting that there are no vulnerability data for Ubuntu 17.04 and 17.10 distributions in the OVAL database because these distributions have reached end of life, meaning that images with these distributions cannot be scanned with Vuls.

**Table 1.** Vulnerability databases used by scanners for different OS distributions

OS	Anchore	Vuls	Clair
Alpine	Alpine-SecDB	Alpine-SecDB	Alpine-SecDB
CentOS	Red Hat OVAL Database	Red Hat OVAL Database and Red Hat Security Advisories	Red Hat Security Data
Debian	Debian Security Bug Tracker	Debian OVAL Database and Debian Security Bug Tracker	Debian Security Bug Tracker
Ubuntu	Ubuntu CVE Tracker	Ubuntu OVAL Database	Ubuntu CVE Tracker

All scanners also refer to the National Vulnerability Database for vulnerability metadata.

For CentOS images, Anchore and Clair report scanning results using Red Hat Security Advisory (RHSA) identifiers, whereas Vuls uses the Common Vulnerabilities and Exposures (CVE) identifiers used in OVAL. We mapped RHSA identifiers to corresponding CVE identifiers, to allow for a comparison between scanners.

Different vulnerabilities may be reported by scanners if scanning experiments take place on different dates. To avoid such discrepancies, we froze the vulnerability databases used by these scanners as of 25 September 2019.

### Image update

A first approach to reduce the number of vulnerabilities in container images is to update their packages to the latest version available in the OS distribution. To study the effect of such updates, we created a script, available in the GitHub repository associated with this paper [18] (see Section “Availability and Requirements"), to identify the package manager in the image, and invoke it to update all OS packages. We updated images on 5 November 2019.

### Image minification

A second approach to reduce the number of vulnerabilities in the images is to remove unnecessary packages, an operation potentially specific to each analysis. Although vulnerabilities in unused packages could not be directly exploited when running the analysis provided by the container image, they still increase the risk of escalation attacks and should therefore be avoided.

We used the open-source ReproZip tool [19] to capture the list of packages used by an analysis. ReproZip first captures the list of files involved in the analysis, through system call interception, then retrieves the list of associated software packages by querying the package manager. We extend this list with a passlist of packages required for the system to function, such as coreutils and bash, and with all the dependencies of the required packages, retrieved using Debtree. Repoquery [20] could be used in RPM-based distributions instead. Our minification script, available in the GitHub repository associated with this paper [18] (see Section “Availability and Requirements"), , installs ReproZip in the image to minify, runs an analysis to collect a ReproZip trace, and finally deletes all unnecessary packages. We had used the Neurodocker tool [21] initially, but it did not affect the detected vulnerabilities as it was removing unused files without using the package manager.

Image minification is a tedious operation because it requires (i) creating relevant analysis examples and (ii) running these examples in the container image to identify the packages required by the application. The resulting minified container image is only valid for the examples selected in the minification process because other executions might require a different set of packages.

While the Boutiques specification allows developers to specify analysis tests, we found that this feature was not consistently used in the studied container images. Therefore, we relied on analysis examples found in the documentation of the applications. Using this approach, we minified 5 Debian- or Ubuntu-based BIDS app images.

## Results

**Figure 1:**
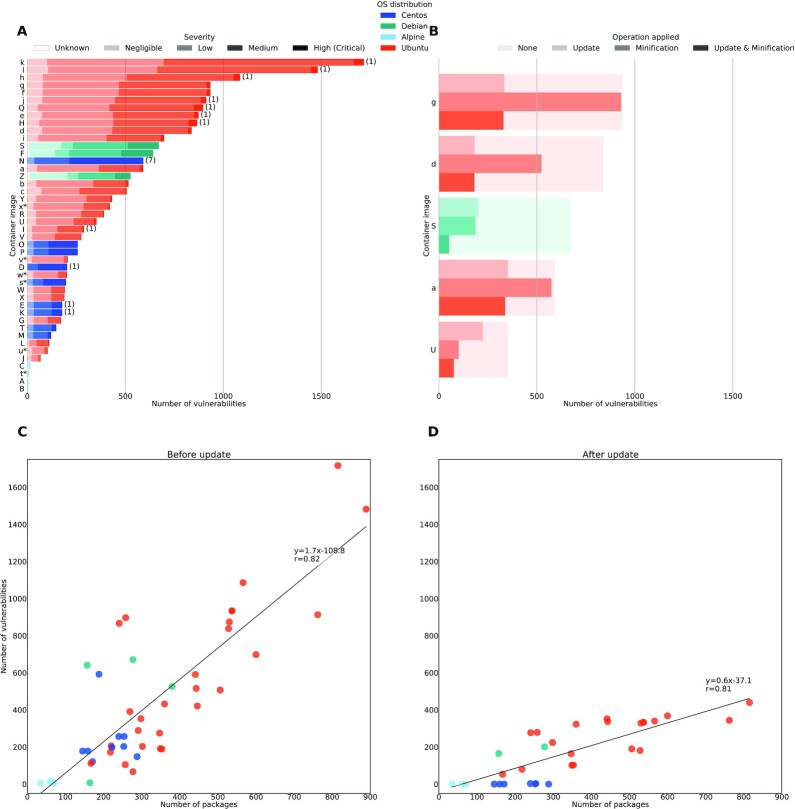
Number of vulnerabilities detected by Anchore and Stools in container images. (a) Number of vulnerabilities by container image and severity, showing hundreds of detected vulnerabilities per image. Images s*, t*, u*, v*, w*, and x* are Singularity images scanned by Stools, and others are Docker images scanned using Anchore. High and critical vulnerabilities are represented with the same solid color and critical vulnerabilities are reported in paretheses. (b) Effect of image minification and package update on 5 container images, showing that both techniques are complementary. (c) Number of vulnerabilities by number of packages, showing a strong linear relationship. (d) Number of vulnerabilities by number of packages after package update, showing that software updates importantly reduce the number of detected vulnerabilities.

Figure 1 presents our results. All the collected data are available in our GitHub repository at https://github.com/big-data-lab-team/container-vulnerabilities-paper with a Jupyter notebook to regenerate the figures.

### Detected vulnerabilities

An important amount of vulnerabilities were found in the tested container images (Fig. 1a), with a mean of 460 vulnerabilities per image and a median of 321. Moreover, a significant fraction of detected vulnerabilities are of high severity (CVSS score ≥7.0) and a few of them are of critical severity (CVSS ≥9.0). Remote attackers could possibly exploit these vulnerabilities to execute arbitrary code in the container or to store arbitrary files in the system. For instance, vulnerability CVE-2019-3462 could lead to remote code execution through human-in-the-middle attacks and vulnerability CVE-2018-1000802 could lead to arbitrary file injections in the file system through unfiltered Python user input. Among other consequences, remote code execution and injection of arbitrary files could be leveraged to steal user credentials, to use CPU cycles or storage space for illegitimate purposes, or to attempt denial-of-service attacks against the system.

Images based on the Alpine distribution had the fewest vulnerabilities, but no significant difference in the numbers of vulnerabilities detected in Ubuntu, Debian, or CentOS distributions was observed.

Unsurprisingly, a strong linear relationship is found between the number of detected vulnerabilities and the number of packages present in the image (Fig. 1c, *r* = 0.82, *P* < 10^−11^). On average, 1.7 vulnerabilities are introduced for each new package installation. This observation motivates a systematic review of software dependencies by application developers, to avoid unnecessary packages in container images. Compared to Ubuntu and Debian distributions, CentOS images seem to have fewer vulnerabilities by package on average, although data are too scarce to draw conclusions.

### Effect of image update

Updating container images reduces the number of vulnerabilities by package by a factor of 3 on average, resulting in only 0.6 extra vulnerabilities by package (Fig. 1d, *r* = 0.81, *P* < 10^−7^). Twelve container images are missing on this figure. Six of them are Singularity images that we did not update, and 6 of them could not be updated by our script owing to the following issues. One image was built from base Docker image CentOS 7.1.1503, which includes package fakesystemd conflicting with several other distribution packages: updating would require either updating the base image or swapping package fakesystemd for systemd. Three images were built from Ubuntu 17.04 or Debian 8, which have reached end of life: updating would require changing the source list to make it point to old releases. Two images could not be updated because some files previously installed through the package manager had been removed by other means, leading to failure of the update process. Software updates did not break the tested analyses in the remaining images. We did not investigate the potential consequences of image updates on numerical stability because it would require a full separate study. Updating packages seems to be an efficient way to avoid vulnerabilities. It is not an ultimate solution, however, because a substantial number of vulnerabilities remain.

### Effect of minification

Using the ReproZip-based approach described previously, we minified 5 different images covering the spectrum of detected vulnerabilities (Fig. 1b). We find that minification reduces the number of vulnerabilities, albeit less systematically than package update. For some container images, such as Image S, minification removes >70% of the detected vulnerabilities. For other images, such as Image g, it only reduces the number of vulnerabilities by <1%. The effect of minification stems from the number of packages that can be removed, which varies greatly across images. For instance, Images g and a have a large number of packages, but almost all of them are required by the analysis, which makes minification less useful. In other cases, a limited number of unnecessary packages contain a significant number of vulnerabilities, which makes minification very effective. This was the case in Images d, S, and U, where removing compilers and kernel headers reduced the number of vulnerabilities by an important fraction. Minification did not create any errors in these images because all the dependencies were taken care of properly. Common packages removed by the minification process were compilers, unused Python packages, and unused file compression utilities.

### Combined effect of image update and minification

Package update and image minification remove different types of vulnerabilities. The former is efficient against vulnerabilities that have been fixed by package maintainers, while the latter targets unused software. In 2 of the 5 tested images (Images S and U), we find that combining update and minification further reduces the number of vulnerabilities compared to using only 1 of these processes (Fig. 1b).

### Differences between scanners

**Figure 2:**
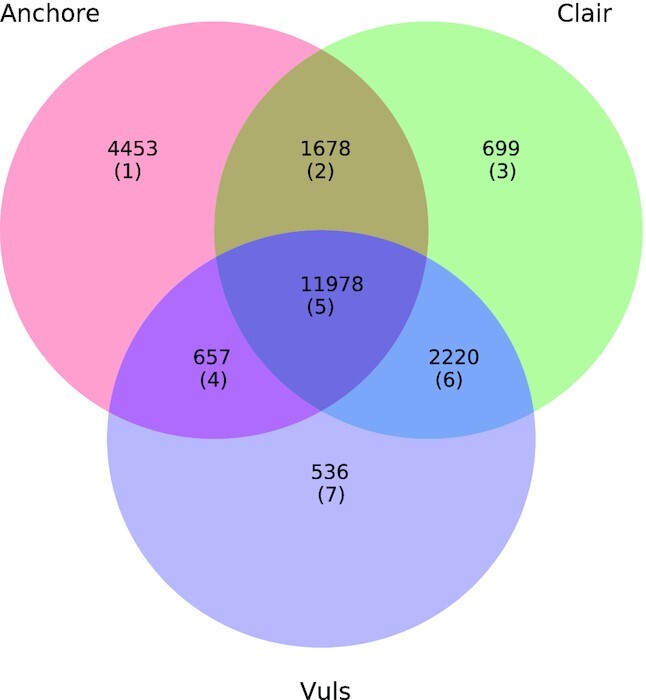
Differences between vulnerabilities detected by the different scanners. The Jaccard coefficients between the sets of detected vulnerabilities are quite low, showing important discrepancies between the scanners: Jaccard(Anchore, Clair) = 0.63, Jaccard(Anchore, Vuls) = 0.59, Jaccard(Vuls, Clair) = 0.80. Two Ubuntu 17.04 images were not included in this comparison because they cannot be scanned by Vuls.

The results presented so far were obtained with Anchore (Docker images) and Stools (Singularity images). We scanned the Docker images with 2 other tools, Clair and Vuls, to evaluate the stability of our results. Important discrepancies were found between scanners (Fig. 2), in particular between Anchore and the other 2 scanners, for which Jaccard coefficients as low as 0.6 were found, meaning that scanning results only overlapped by 60%. Vuls and Clair seem to be in better agreement, with a Jaccard coefficient of 0.8.

We analyzed these results and explained some reasons behind the observed discrepancies. Of 4,453 vulnerabilities detected by Anchore only (Region 1 in Fig. 2), 4,443 are found in the development package of the C library (linux-libc-dev in Ubuntu and Debian). Clair detects only Debian vulnerabilities in linux-libc-dev, whereas Vuls does not detect vulnerabilities in this package at all. Because Anchore ignores Debian vulnerabilities flagged as “minor", it might either detect (Region 2) or ignore (Region 3) the Debian vulnerabilities detected by Clair in linux-libc-dev. The remaining 10 vulnerabilities in Region 1 are found in sub-packages of vulnerable packages: they are correctly reported by Anchore and missed by Vuls and Clair.

Many vulnerabilities in Regions 3 and 4 are from images based on Ubuntu 14.04. In the Ubuntu CVE tracker database used by Clair and Anchore, there are 2 entries for Ubuntu 14.04: 1 for LTS (Long-Term Support), a Ubuntu release with 5 years of technical support, and another 1 for ESM (Extended Security Maintenance), a release that provides security patches beyond the 5 years covered by LTS. Although all the scanned images are LTS, Clair refers to the ESM database entry while Anchore and Vuls refer to the LTS database entry. The vulnerabilities present in Region 3 due to this discrepancy are incorrectly missed by Anchore and Vuls: they have been detected in ESM but were already present in LTS. The vulnerabilities in Region 4 are incorrectly missed by Clair: they have been fixed in ESM but are still present in LTS.

Some vulnerabilities in Region 6 are due to bugs in Anchore: the “epoch bug" ignores vulnerabilities related to package versions that contain an epoch (:); the “out of standard bug" ignores vulnerabilities that are ignored by the Ubuntu distribution. We reported these bugs to the Anchore developers through their Slack channel. Some vulnerabilities in Region 6 are also due to the fact that Anchore intentionally ignores Debian vulnerabilities flagged as “minor".

Finally, 32 vulnerabilities that are flagged temporary by the Debian distribution are reported by Vuls but not by Anchore or Clair (Region 7). The remaining 504 vulnerabilities in this region are all found in CentOS images. We were not able to explain why they were detected by Vuls only.

## Discussion

There is a widespread issue with security vulnerabilities in container images used for neuroimaging analyses, and it is likely to affect other scientific disciplines as well. As shown in our results, it is common for container images to hold hundreds of vulnerabilities, including several of critical severity. Container images are affected regardless of the type of analyses that they support, and the main OS distributions Ubuntu, Debian, and CentOS are all affected.

Software updates remove roughly two-thirds of the vulnerabilities found and should certainly be considered the primary solution to this problem. However, in neuroimaging as in other disciplines, software updates are generally discouraged because they can affect analysis results by introducing numerical perturbations in the computations [5,6]. We believe that this position is not viable from an IT security perspective and that it could endanger the entire Big Data processing infrastructure. Instead, we advocate a more systematic analysis of the numerical schemes involved in data analyses, which, coupled with software testing, would make the analyses robust to software updates. As a first step, the packages affecting the analyses could be specifically identified and the others updated, which would largely remove vulnerabilities.

Ultimately, software updates should even occur at runtime rather than when the container image is built. Indeed, it is likely that container images used for scientific data analyses are built only occasionally, perhaps every few weeks when a release becomes available, which may not be compatible with the frequency of required security updates. In fact, there is no definite reason for the application software release cycle to be synchronized with security updates, and security updates should not be dependent on application software developers. Instead, we think it would be relevant for analytics engines to (i) systematically apply security updates when containers start and (ii) run software tests provided by application developers, including numerical tests, before running analyses.

Implementing such a workflow, however, requires a long-term endeavour to evaluate broadly the stability of data analysis pipelines, and to develop the associated software tests. For the shorter term, we identified the following recommendations for application developers to reduce the number of security vulnerabilities in container images:

- Introduce software dependencies cautiously. Software dependencies come with a potential security toll that is often neglected. For instance, it can be tempting to add a complete toolbox to implement a relatively minor operation in a data analysis pipeline, such as a data format conversion, while the same functionality might be available in the existing dependencies of the pipeline, albeit in a less convenient way. Some package managers such as apt also support “weak” package dependencies that are recommended but not necessarily required for installation. Installation of such suggested packages can be avoided using specific options of the package manager (--no-install-recommends in the case of apt). Using lightweight base OS images such as Alpine Linux can also reduce the number of unnecessary dependencies. However, developers should ensure that this does not lead to installing extra dependencies without using the package manager, as mentioned in Point (iv) below.
- Minify container images. Minifying container images is another way to reduce software dependencies. However, the automated minification process that we used in our study is unwieldy for routine use because it requires capturing execution traces with ReproZip to reconstruct the graph of package dependencies required for the analysis. In practice, it would be more practical for software developers to identify and remove unnecessary dependencies when they build containers, based on their knowledge of the application.
- Use OS releases with long-term support. Security updates are not provided for OS distributions that have reached end of life. When a given release of a data analysis pipeline is expected to be used over a long period, typically several years as is common in neurosciences, the life cycle of the distribution release should be considered when choosing a base container image. OS distributions have very different life cycle durations because long and short life cycles serve different purposes. For instance, among Red Hat–based distributions, Fedora releases a new version every 6 months and provides maintenance for ∼1 year, while CentOS releases every 3–5 years and provides maintenance for 10 years. Similarly, Ubuntu and Debian LTS (long-term support) distributions provide security updates for ≥5 years.
- Install packages, not files. Regardless of their support status, base OS distributions covered by the previous recommendation rarely include scientific software. Because vulnerability scanners detect vulnerabilities from the list of installed packages, vulnerabilities contained in files installed without the package manger remain undetected. To reduce such occurrences, scientific software should as much as possible be linked dynamically against libraries provided by the base OS distribution. The use of domain-specific repositories such as NeuroDebian or NeuroFedora in neuroimaging is also useful in this respect because these distributions facilitate transparent software updates and might be covered by image scanners in the future.
- Run image scanners during continuous integration. Scanning container images can be a cumbersome process that could be asynchronously executed during continuous integration (CI), through tools such as Travis CI or Circle CI. Including security scans in CI also allows developers to identify vulnerabilities quickly, before new software versions are released. The Anchore documentation includes specific instructions on how to do so [22]. The Stools repository also includes a Travis CI file that can be reused for this purpose.

Describing specific attacks that would exploit vulnerabilities in container images is out of the scope of our study. We believe that such attacks are likely to exist given that critical vulnerabilities allowing for arbitrary code execution or file injection were found. Attacking systems through containers remains challenging owing to their relative isolation from the host system. Under the assumption that container host users can be trusted, attackers would have to be remote, either in the same network or on a remote network. Two main types of attacks can be envisaged in these conditions: network-based attacks, exploiting vulnerabilities in network clients installed in the container; and data-based attacks, exploiting vulnerabilities through the processing of malicious data injected through third-party systems.

Several types of escalation attacks could be envisaged once remote attackers gain access to the container, in particular related to (i) stealing user credentials; (ii) using the resources allocated to the container for malicious use, resulting in denial of service; and (3) attacking a host network service, e.g., a locally accessible server or a file system daemon. Exploits in the host kernel to break out of the container are always possible but unlikely assuming that the host system is maintained by professional system administrators.

## Conclusion

Most container images used in scientific data analyses contain hundreds of security vulnerabilities, many of which are critical. In the short term, application software developers can address this issue by (i) reducing software dependencies and (ii) applying regular security updates, which requires using OS distributions with long-term support. In the longer term, data analysis pipelines would benefit from in-depth stability analysis to ensure that analytical results are not affected by security updates.

This conclusion is not an alarming message urging system administrators to ban containers from their systems. User-controlled container images are just one of many end-user artifacts that could serve as attack vectors, and to our knowledge no attack has been described to exploit them. More traditional types of attacks targeting user credentials or network connections are likely to remain more common.

## Availability and Requirements

The data and scripts used in this study are available on GitHub with a Jupyter notebook to regenerate the figures:

Project name: container-vulnerabilities-paper

Project home page: https://github.com/big-data-lab-team/container-vulnerabilities-paper

Operating system: Platform independent

Programming language: Python

Other requirements: Jupyter ≥1.0.0, Matplotlib ≥3.3.0, NumPy ≥1.19.1, Pandas ≥1.0.5, SciPy ≥1.6.0

License: GNU General Public License v3.0

## Data Availability

The data underlying this article are available in [23].

## Abbreviations

API: Application Programming Interface; CI: continuous integration; CPU: central processing unit; CVE: Common Vulnerabilities and Exposures; OS: operating system; OVAL: Open Vulnerability and Assessment Language; RHSA: Red Hat Security Advisory.

## Competing Interests

The authors declare that they have no competing interests.

## Authors' Contributions

BK: Investigation, Software, Methodology, Writing - Original Draft Preparation, Validation, Visualization; MD: Software; AH: Conceptualization, Supervision, Methodology, Validation, Writing - Review & Editing; TG: Conceptualization, Supervision, Methodology, Validation, Writing - Review & Editing, Visualization, Funding Acquisition.

## Supplementary Material

giab025_GIGA-D-20-00311_Original_Submission

giab025_GIGA-D-20-00311_Revision_1

giab025_GIGA-D-20-00311_Revision_2

giab025_Response_to_Reviewer_Comments_Original_Submission

giab025_Response_to_Reviewer_Comments_Revision_1

giab025_Reviewer_1_Report_Original_Submission Chris Armit -- 11/11/2020 Reviewed

giab025_Reviewer_2_Report_Original_Submission Erik C. Johnson, Ph.D. -- 11/14/2020 Reviewed

giab025_Reviewer_3_Report_Original_Submission Yaroslav Halchenko -- 11/30/2020 Reviewed
