# Supplementary material for: An analysis of security vulnerabilities in container images for scientific data analysis
Source: Gigascience. 2021 Jun 3;10(6):giab025. doi: 10.1093/gigascience/giab025 (PMC8173661; doi:10.1093/gigascience/giab025)
Supplement: giab025_GIGA-D-20-00311_Original_Submission [file giab025_giga-d-20-00311_original_submission.pdf]

GigaScience

An Analysis of Security Vulnerabilities in Container Images for Scientific Data Analysis

--Manuscript Draft--

**Manuscript Number:**	GIGA-D-20-00311
**Full Title:**	An Analysis of Security Vulnerabilities in Container Images for Scientific Data Analysis
**Article Type:**	Technical Note
**Funding Information:**	Canada Research Chairs — Dr Tristan Glatard<br>Fonds de Recherche du Québec - Nature et Technologies — Dr Tristan Glatard
**Abstract:**	Software containers greatly facilitate the deployment and reproducibility of scientific data analyses on high-performance computing clusters (HPC). However, container images often contain outdated or unnecessary software packages, which increases the number of security vulnerabilities in the images and widens the attack surface of the infrastructure. This paper presents a vulnerability analysis of container images for scientific data analysis. We compare results obtained with four vulnerability scanners, focusing on the use case of neuroscience data analysis, and quantifying the effect of image update and minification on the number of vulnerabilities. We find that container images used for neuroscience data analysis contain hundreds of vulnerabilities, that software updates remove about two thirds of these vulnerabilities, and that removing unused packages is also effective. We conclude with recommendations on how to build container images with a reduced amount of vulnerabilities.
**Corresponding Author:**	Tristan Glatard<br><br>CANADA
**Corresponding Author Secondary Information:**	
**Corresponding Author's Institution:**	
**Corresponding Author's Secondary Institution:**	
**First Author:**	Bhupinder Kaur
**First Author Secondary Information:**	
**Order of Authors:**	Bhupinder Kaur<br>Mathieu Dugré<br>Aiman Hanna<br>Tristan Glatard
**Order of Authors Secondary Information:**	
**Additional Information:**	
**Question**	**Response**
Are you submitting this manuscript to a special series or article collection?	No
**Experimental design and statistics**<br><br>Full details of the experimental design and statistical methods used should be given in the Methods section, as detailed in our Minimum Standards Reporting Checklist.	Yes

Powered by Editorial Manager® and ProduXion Manager® from Aries Systems Corporation

Information essential to interpreting the data presented should be made available in the figure legends.<br><br>Have you included all the information requested in your manuscript?	
**Resources**<br><br>A description of all resources used, including antibodies, cell lines, animals and software tools, with enough information to allow them to be uniquely identified, should be included in the Methods section. Authors are strongly encouraged to cite Research Resource Identifiers (RRIDs) for antibodies, model organisms and tools, where possible.<br><br>Have you included the information requested as detailed in our Minimum Standards Reporting Checklist?	Yes
**Availability of data and materials**<br><br>All datasets and code on which the conclusions of the paper rely must be either included in your submission or deposited in publicly available repositories (where available and ethically appropriate), referencing such data using a unique identifier in the references and in the "Availability of Data and Materials" section of your manuscript.<br><br>Have you have met the above requirement as detailed in our Minimum Standards Reporting Checklist?	Yes

Powered by Editorial Manager® and ProduXion Manager® from Aries Systems Corporation

Manuscript
Click here to access/download;Manuscript;paper.pdf

Placeholder for OUP logo oup.pdf

GigaScience, 20xx, 1–6

doi: xx.xxxx/xxxx
Manuscript in Preparation

An Analysis of Security Vulnerabilities in Container Images for Scientific Data Analysis

Bhupinder Kaur[1], Mathieu Dugré[1], Aiman Hanna[1] and Tristan Glatard[1]

[1]Department of Computer Science and Software Engineering, Concordia University, Montreal, Canada

Abstract

Software containers greatly facilitate the deployment and reproducibility of scientific data analyses on high-performance computing clusters (HPC). However, container images often contain outdated or unnecessary software packages, which increases the number of security vulnerabilities in the images and widens the attack surface of the infrastructure. This paper presents a vulnerability analysis of container images for scientific data analysis. We compare results obtained with four vulnerability scanners, focusing on the use case of neuroscience data analysis, and quantifying the effect of image update and minification on the number of vulnerabilities. We find that container images used for neuroscience data analysis contain hundreds of vulnerabilities, that software updates remove about two thirds of these vulnerabilities, and that removing unused packages is also effective. We conclude with recommendations on how to build container images with a reduced amount of vulnerabilities.

Key words: Containers; Docker; Singularity; Security Vulnerabilities; Neuroimaging.

Introduction

Software containers have emerged has an efficient solution to deploy scientific data analyses on HPC clusters, due to their portability, ease of use, and limited overhead. On HPC systems, the Singularity [1] framework is often preferred to Docker due to its secure handling of multi-user environments and convenient support for Docker images. Singularity is now available in dozens of HPC clusters around the world and routinely used for Big Data analysis.

Taking advantage of core Linux kernel features such as namespaces, control groups and chroot, containers isolate processes from the host computer, and control the memory, CPU, network and file-system resources assigned to them. However, containers still share the kernel, mounted file systems and some devices with the host, which raises security concerns [2, 3, 4] and opens the door to privilege escalation, denial of service, information leak and other types of attacks [5].

Container images typically include full operating system (OS) distributions in addition to data analysis software and their dependencies. They are rarely updated due to concerns that software updates will interfere with the results [6, 7]. Images also typically include more dependencies than required, to make them easier to reuse between experiments. As a result, over 30% of official images in DockerHub have been shown to contain high-priority security vulnerabilities [8], images on average contain over 180 vulnerabilities [9], and vulnerabilities are often caused by outdated packages [10].

In this study, we focus on the vulnerabilities present in container images deployed on HPC clusters for scientific data analysis, in particular in the neuroimaging domain. We address the following questions:

What is the current amount of vulnerabilities in container images deployed on HPC clusters? Vulnerabilities are possible attack vectors that can seriously compromise the security of HPC clusters and the integrity of user data. We report vulnerability scans produced by four popular image scanning tools: Anchore, Vuls, Clair, and Singularity tools.

Can the amount of vulnerabilities be reduced by updating the images? For many reasons related to reproducibility and the life-cycle of research projects, container images deployed on HPC clusters often include outdated software. We report on the effect of software updates on the amount of vulnerabilities found in images.

Can the amount of vulnerabilities be reduced by minifying images? Container images often include more software packages than necessary for a typical analysis. We report on the impact of unused software packages on the presence of vulnerabilities.

The remainder of this paper describes the container images and scanners used in our experiment, and our methodology for updating and minifying images. Results present the vulnerabilities detected in container images, quantify the effectiveness of updating and minifying images, and explain the differences observed between scanners. In conclusion, we provide a set of image creation guidelines for a more secure deployment of containers on HPC clusters.

OS	Anchore	Vuls	Clair
**Alpine**	Alpine-SecDB	Alpine-SecDB	Alpine-SecDB
**CentOS**	Red Hat OVAL Database	Red Hat OVAL Database and Red Hat Security Advisories	Red Hat Security Data
**Debian**	Debian Security Bug Tracker	Debian OVAL Database and Debian Security Bug Tracker	Debian Security Bug Tracker
**Ubuntu**	Ubuntu CVE Tracker	Ubuntu OVAL Database	Ubuntu CVE Tracker

Table 1. Vulnerability databases used by scanners for different OS distributions. All scanners also refer to the National Vulnerability Database (NVD) for vulnerability metadata.

Materials and Tools

We used container images from two popular application frameworks, as well as four of the major image scanners.

Container Images

We scanned all container images available at the time of this study on two containerization frameworks used in neuroscience: BIDS apps [11] (26 images) and Boutiques [12] (18 images), totalling 44 container images. At the time of the study, BIDS apps had 27 images, out of which one wasn't available on DockerHub. Boutiques had 49 images, however, only 23 unique images were listed, out of which 3 couldn't be retrieved and 2 were already included in BIDS apps. All the final 26 images from BIDS apps were Docker images, whereas the 18 Boutiques images contained 12 Docker images and 6 Singularity images.

Image Scanners

We compared the results obtained with four container image scanners: Anchore, Vuls, and Clair to scan Docker images, and Singularity Container Tools (Stools) to scan Singularity images.

Anchore is an end-to-end, open-source container security platform. It analyzes container images and lists vulnerable OS packages, non-OS packages (Python, Java, Gem, and npm), and files. In our experiments, we used Anchore Engine version 0.5.0 through Docker image `anchore/anchore-engine:v0.5.0`, and Anchore vulnerability database version 0.0.11.

Vuls is an open-source vulnerability scanner for Linux and FreeBSD. It offers both static and dynamic scanning, and both local and remote scanning. In our experiments, we used Vuls 0.9.0, executed through Docker image `vuls/vuls:0.9.0` in remote dynamic mode.

Clair is an open-source and extensible vulnerability scanner for Docker and appc container images, developed by CoreOS (now Container Linux), a Linux distribution to deploy container clusters. We used Clair through Clair-scanner, a tool to facilitate the testing of container images against a local Clair server. We used Clair version 2.0.6, executed through Docker image `arminc/clair-local-scan:v2.0.6`. For the vulnerability database, we used Docker image `arminc/clair-db:latest`, last updated on 2019-09-18.

Singularity Tools (Stools) are an extension of Clair for Singularity images. Stools exports Singularity images to `tar.gz` format, acting as a single layer Docker image to circumvent the Docker-specific requirements in the Clair API. In our experiments, we used Singularity Tools version 3.2.1 through Docker image `vanessa/stools-clair:v3.2.1`. Since Stools uses Clair internally for scanning, the vulnerability databases used by Stools are the same as mentioned for Clair. To scan Singularity images, we followed the steps mentioned in the Stools documentation.

Vulnerability Databases

Scanners refer to two types of vulnerability databases (Table 1). The first one is the Open Vulnerability and Assessment Language (OVAL) database, an international open standard that supports various OS distributions including Ubuntu, Debian and CentOS but not Alpine. The second one are vulnerability databases from specific OS distributions, such as Alpine-SecDB, Debian Security Bug Tracker, Ubuntu CVE Tracker, or Red Hat Security Data. In these databases, OS distributions often assign a status to each vulnerability, to keep track of required and available security fixes in different versions of the distribution. Vuls uses OVAL databases for all distributions except Alpine. On the contrary, Clair exclusively refers to distribution-specific databases. Anchore uses OVAL only for CentOS, as distribution-specific databases are assumed to be more complete. It is also worth noting that there is no vulnerability data for Ubuntu 17.04 and 17.10 distributions in the OVAL database, since these distributions have reached end of life, meaning that images with these distributions cannot be scanned with Vuls.

For CentOS images, Anchore and Clair give scanning results using Red Hat Security Advisory (RHSA) identifiers, whereas Vuls uses the Common Vulnerabilities and Exposures (CVE) identifiers used in OVAL. We mapped RHSA identifiers to corresponding CVE identifiers, to allow for a comparison between scanners.

Different vulnerabilities may be reported by scanners if scanning experiments take place on different dates. To avoid such discrepancies, we froze the vulnerability databases used by these scanners as of 2019-09-25.

Image Update

A first approach to reduce the number of vulnerabilities in container images is to update their packages to the latest version available in the OS distribution. To study the effect of such updates, we developed a script (available here) to identify the package manager in the image, and invoke it to update all OS packages. We updated images on 2019-11-05.

Image Minification

A second approach to reduce the number of vulnerabilities in the images is to remove unnecessary packages, an operation potentially specific to each analysis. We used the open-source ReproZip tool [13] to capture the list of packages used by an analysis. ReproZip first captures the list of files involved in the analysis, through system call interception, then retrieves the list of associated software packages, by querying the package manager. We extend this list with a passlist of packages required for the system to function, such as `coreutils` and `bash`, and with all the dependencies of the required packages, retrieved using Debtree. Repoquery could be used in RPM-based distributions instead. Our minification script, available here, installs ReproZip in the image to minify, runs an analysis to collect a ReproZip trace, and finally deletes all unnecessary packages. We had used the Neurodocker tool initially, but it did not affect the detected vulnerabilities as it was removing unused files without using the package manager.

Using this approach, we minified five Debian- or Ubuntu-based BIDS app images, using basic analysis examples found in the applications documentation.

Results

Figure 1 presents our results. All the collected data are available in our GitHub repository at https://github.com/big-data-lab-team/container-vulnerabilities-paper with a Jupyter notebook to regenerate the figures.

Detected Vulnerabilities

An important amount of vulnerabilities were found in the tested container images (Fig 1-**A**), with an average of 460 vulnerabilities per image and a median of 321. In comparison, no vulnerabilities were found in base Docker images `ubuntu:20.04` and `centos:7` after package update. Moreover, a significant fraction of detected vulnerabilities are of high severity (CVSS score >=7.0) and a few of them are of critical severity (CVSS >= 9.0). Remote attackers could possibly exploit these vulnerabilities to execute arbitrary code in the container, by crafting responses to specific network requests. Images based on the Alpine distribution had the lowest numbers of vulnerabilities, but no significant difference in the numbers of vulnerabilities detected in Ubuntu, Debian or CentOS distributions was observed.

Unsurprisingly, a strong linear relationship is found between the number of detected vulnerabilities and the number of packages present in the image (Fig 1-**C**, r=0.82, $p < 10^{-11}$). On average, 1.7 vulnerabilities are introduced for each new package installation. This observation motivates a systematic review of software dependencies by application developers, to avoid unnecessary packages in container images. This is also an argument in favor of lightweight distributions such as Alpine. Compared to Ubuntu and Debian distributions, CentOS images seem to have a lower number of vulnerabilities by package on average, although data is too scarce to conclude.

Effect of image update

Updating container images reduces the number of vulnerabilities by package by a factor of 3 on average, resulting in only 0.6 extra vulnerabilities by package (Fig 1-**D**, r=0.81, $p < 10^{-7}$). Twelve container images are missing on this figure: six of them could not be updated due to various issues with the package manager, and six of them are Singularity images that we didn't update. In spite of the associated reproducibility challenges, updating packages therefore appears to be an efficient way to avoid vulnerabilities. It is not an ultimate solution though, as a substantial number of vulnerabilities remain.

Effect of minification

Another approach to reduce the number of vulnerabilities involves deleting unnecessary packages from the container images. It is a tedious operation, as it requires running an actual data analysis in the container image, to identify the packages required by the application. In addition, the resulting container image is only valid for the specific type of analysis used in the minification process, as other executions might require a different set of packages.

Using the ReproZip-based approach described previously, we minified 5 different images covering the spectrum of detected vulnerabilities (Fig 1-**B**). We find that minification reduces the number of vulnerabilities, albeit less systematically than package update. For some container images, such as image **S**, minification removes more than 70% of the detected vulnerabilities. For other images, such as image **g**, it only reduces the number of vulnerabilities by less than 1%. The effect of minification stems from the number of packages that can be removed, which varies greatly across images. For instance, images **g** and **a** have a large number of packages, but the last majority of them is required by the analysis, which makes minification less useful. In other cases, a limited number of unnecessary packages contain a significant number of vulnerabilities, which makes minification very impactful. This was the case in images **d**, **S** and **U**, where removing compilers and kernel headers reduced the number of vulnerabilities by an important fraction.

Combined effect of image update and minification

Package update and image minification remove different types of vulnerabilities. The former is efficient against vulnerabilities that have been fixed by package maintainers, while the latter targets unused software. In two of the five tested images (images **S** and **U**), we find that combining update and minification further reduces the number of vulnerabilities compared to using only one of these processes (Fig 1-**B**).

Differences between scanners

The results presented so far were obtained with Anchore (Docker images) and Stools (Singularity images). We scanned the Docker images with two other tools, Clair and Vuls, to evaluate the stability of our results. Important discrepancies were found between scanners (Fig 2), in particular between Anchore and the other two scanners, for which Jaccard coefficients as low as 0.6 were found, meaning that scanning results only overlapped by 60%. Vuls and Clair appear to be in better agreement, with a Jaccard coefficient of 0.8.

We analyzed these results and explained some reasons behind the observed discrepancies. Out of 4453 vulnerabilities detected by Anchore only (region **1** in Fig 2), 4443 are found in the development package of the C library (`linux-libc-dev` in Ubuntu and Debian). Clair detects only Debian vulnerabilities in `linux-libc-dev`, whereas Vuls do not detect vulnerabilities in this package at all. Since Anchore ignores Debian vulnerabilities flagged as `minor`, it might either detect (region **2**) or ignore (region **3**) the Debian vulnerabilities detected by Clair in `linux-libc-dev`. The remaining 10 vulnerabilities in region **1** are found in sub-packages of vulnerable packages: they are correctly reported by Anchore and missed by Vuls and Clair.

Many vulnerabilities in region **3** and **4** are from images based on Ubuntu 14.04. In the Ubuntu CVE tracker database used by Clair and Anchore, there are two entries for Ubuntu 14.04: one for LTS (Long-Term Support), a Ubuntu release with 5 years of technical support, and another one for ESM (Extended Security Maintenance), a release that provides security patches beyond the 5 years covered by LTS. Although all the scanned images are LTS, Clair refers to the ESM database entry while Anchore and Vuls refer to the LTS database entry. The vulnerabilities present in region **3** due to this discrepancy are incorrectly missed by Anchore and Vuls: they have been detected in ESM but were already present in LTS. The vulnerabilities in region **4** are incorrectly missed by Clair: they have been fixed in ESM but are still present in LTS.

Some vulnerabilities in region **6** are due to bugs in Anchore: the *epoch bug* ignores vulnerabilities related to package versions that contain an epoch (`:`); the *out of standard bug* ignores vulnerabilities that are ignored by the Ubuntu distribution. We reported these bugs to the Anchore developers through their Slack channel. Some vulnerabilities in region **6** are also due to the fact that Anchore intentionally ignores Debian vulnerabilities flagged as `minor`.

Finally, 32 vulnerabilities that are flagged temporary by the

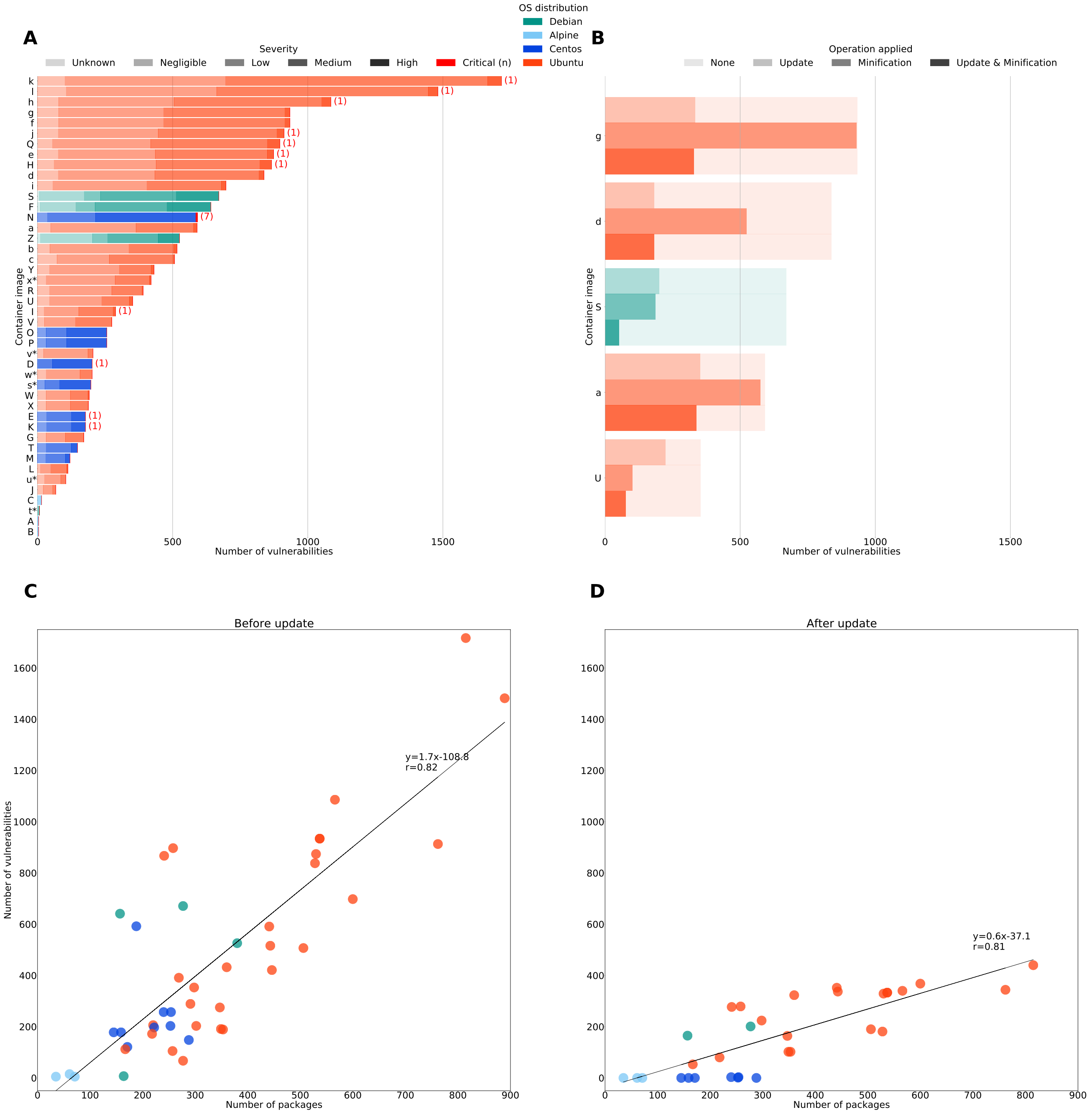

Figure 1. Number of vulnerabilities detected by Anchore and Stools in container images. **(A)** Number of vulnerabilities by container image and severity, showing hundreds of detected vulnerabilities per image. Images s*,t*,u*,v*,w* and x* are Singularity images scanned by Stools and others are Docker images scanned using Anchore. **(B)** Effect of image minification and package update on 5 container images, showing that both techniques are complementary **(C)** Number of vulnerabilities by number of packages, showing a strong linear relationship. **(D)** Number of vulnerabilities by number of packages *after package update*, showing that software updates importantly reduce the number of detected vulnerabilities.

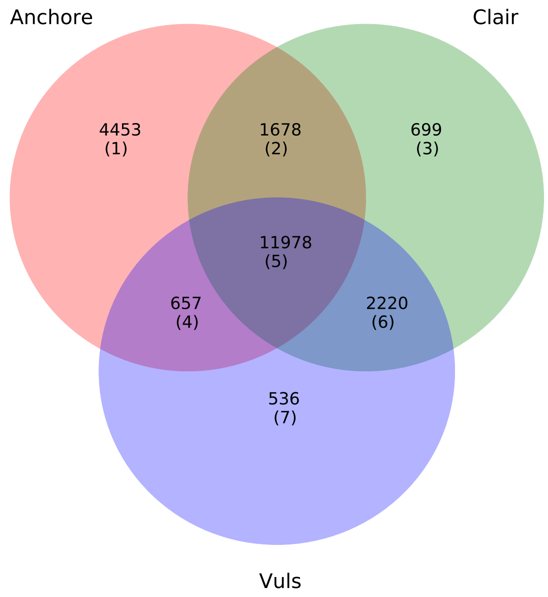

Figure 2. Differences between vulnerabilities detected by the different scanners. The Jaccard coefficients between the sets of detected vulnerabilities are quite low, showing important discrepancies between the scanners: Jaccard(Anchore, Clair) = 0.63, Jaccard(Anchore, Vuls) = 0.59, Jaccard(Vuls, Clair) = 0.80. Two Ubuntu 17.04 images weren't included in this comparison as they cannot be scanned by Vuls.

Debian distribution are reported by Vuls but not by Anchore or Clair (region **7**). The remaining 504 vulnerabilities in this region are all found in CentOS images. We weren't able to explain why they were detected by Vuls only.

Discussion

There is a widespread issue with security vulnerabilities in container images used for neuroimaging analyses, and it is likely to impact other scientific disciplines as well. As shown in our results, it is common for container images to hold hundreds of vulnerabilities, including several of critical severity. Container images are impacted regardless of the type of analyses that they support, and the main OS distributions Ubuntu, Debian and CentOS are all affected.

Software updates remove about two-thirds of the vulnerabilities found and should certainly be considered the primary solution to this problem. However, in neuroimaging as in other disciplines, software updates are generally discouraged because they can affect analysis results by introducing numerical perturbations in the computations [6, 7]. We believe that this position is not viable from an IT security perspective, and that it could endanger the entire Big Data processing infrastructure, starting with the HPC centers. Instead, we advocate a more systematic analysis of the numerical schemes involved in data analyses, which, coupled with software testing, would make the analyses robust to software updates. As a first step, the packages impacting the analyses could be specifically identified and the others updated, which would largely remove vulnerabilities.

Ultimately, software updates should even occur at runtime rather than when the container image is built. Indeed, it is likely that container images used for scientific data analyses be built only occasionally, perhaps every few weeks when a release becomes available, which may not be compatible with the frequency of required security updates. In fact, there is no definite reason for the application software release cycle to be synchronized with security updates, and security updates shouldn't be dependent on application software developers. Instead, we think it would be relevant for analytics engines to (1) systematically apply security updates when containers start, and (2) run software tests provided by application developers, including numerical tests, before running analyses.

Implementing such a workflow, however, requires a long-term endeavour to evaluate broadly the stability of data analysis pipelines, and to develop the associated software tests. For the shorter term, we identified the following recommendations for application developers to reduce the number of security vulnerabilities in container images:

i. *Introduce software dependencies cautiously*. Software dependencies come with a potential security toll that is often neglected. For instance, it can be tempting to add a complete toolbox to implement a relatively minor operation in a data analysis pipeline, such as a data format conversion, while the same functionality might be available in the existing dependencies of the pipeline, albeit in a less convenient way.

ii. *Use lightweight base images such as Alpine Linux*. Base images often come with packages that are useful in personal computers or servers, but not in containers dedicated to a specific data analysis. In addition, lightweight distributions define packages with a fine granularity, allowing developers to avoid installing unnecessary dependencies. For instance, the compressed size of the Alpine Linux base image on DockerHub is only 2.7 MB while that of the Ubuntu base image is 27 MB.

iii. *Use OS releases with long-term support.* Security updates are not provided for OS distributions that reached end of life. When a given release of a data analysis pipeline is expected to be used over a long period of time, typically several years as it is common in neurosciences, the life cycle of the distribution release should be considered when choosing a base container image. OS distributions have very different life cycle durations, as long and short life cycles serve different purposes. For instance, among RedHat-based distributions, Fedora release a new version every 6 months and provide maintenance for about a year, while CentOS release every 3-5 years and provide maintenance for 10 years. Similarly, Ubuntu LTS (long-term support) distributions provide free security updates for 5 years, and Debian stable releases are maintained for 3 years.

iv. *Install packages, not files*. Vulnerability scanners such as Anchore, Clair or Vuls detect vulnerabilities from the list of packages installed in a container image. Therefore, vulnerabilities contained in software tools installed through direct file download rather than through the package manager would go completely undetected. Domain-specific distributions such as NeuroDebian or NeuroFedora in neuroimaging are useful in this respect.

v. *Minify container images.* The automated minification process that we used in our study is unwieldy for a routine use, as it requires capturing execution traces with ReproZip to reconstruct the graph of package dependencies required for the analysis. In practice, it would be more practical for software developers to identify and remove unnecessary dependencies when they build containers, based on their knowledge of the application.

vi. *Run image scanners during continuous integration.* Scanning container images can be a cumbersome process that could be asynchronously executed during continuous integration (CI), through tools such as Travis CI or Circle CI. Including security scans in CI also allows developers to identify vulnerabilities quickly, before new software versions are released.

Describing specific attacks against HPC systems that would exploit vulnerabilities in container images is out of the scope of our study. We believe that such attacks are likely to exist, al-

though attacking HPC systems through containers is challenging due to their relative isolation from the host system. First, under the assumption that legitimate HPC users can be trusted, attackers would have to be remote to the container, either in the same network or on a remote network. Two main types of attacks can be envisaged in these conditions: network-based attacks, exploiting vulnerabilities in network clients installed in the container, and data-based attacks, exploiting vulnerabilities through the processing of malicious data injected through third-party systems.

Several types of escalation attacks could be envisaged once remote attackers gain access to the container, in particular related to (1) using the resources allocated to the container for malicious use, such as storing data in the file system or using CPU cycles, resulting in denial of service for the user running the container and possibly for other HPC users, and (2) attacking a host network service, for instance a scheduler or a file system daemon. Exploits in the host kernel to break out of the container are always possible but unlikely assuming that the host system is maintained by professional system administrators.

Conclusion

Most container images deployed on HPC clusters for scientific data analyses contain hundreds of security vulnerabilities, many of which are critical. In the short term, application software developers can address this issue by: (1) minifying container images, by using lightweight OS distributions and reducing software dependencies, and (2) applying regular security updates, which requires using OS distributions with long-term support. Longer term, data analysis pipelines would benefit from in-depth stability analysis, to ensure that analytical results are not affected by security updates.

This conclusion is not an alarming message urging HPC administrators to ban containers from their systems. User-controlled container images are just one of many end-user artifacts that could serve as attack vectors, and to our knowledge no attack has been described to exploit them. More traditional types of attacks targeting user credentials or network connections are likely to remain more common.

Availability of Data and Materials

The data and scripts used in this study are available in the GitHub repository at https://github.com/big-data-lab-team/container-vulnerabilities-paper with a Jupyter notebook to regenerate the figures. This manuscript is based on version 0.1 of the repository, available as DOI 10.5281/zenodo.4136599.

Competing interests

The author(s) declare(s) that they have no competing interests.

References

1. Kurtzer GM, Sochat V, Bauer MW. Singularity: Scientific containers for mobility of compute. PloS one 2017;12(5):e0177459.
2. Martin A, Raponi S, Combe T, Di Pietro R. Docker ecosystem–vulnerability analysis. Computer Communications 2018;122:30–43.
3. Sultan S, Ahmad I, Dimitriou T. Container Security: Issues, Challenges, and the Road Ahead. IEEE Access 2019;7:52976–52996.
4. Combe T, Martin A, Di Pietro R. To Docker or not to Docker: A security perspective. IEEE Cloud Computing 2016;3(5):54–62.
5. Gantikow H, Reich C, Knahl M, Clarke N. Providing security in container-based HPC runtime environments. In: International Conference on High Performance Computing; 2016. p. 685–695.
6. Gronenschild EH, Habets P, Jacobs HI, Mengelers R, Rozendaal N, Van Os J, et al. The effects of FreeSurfer version, workstation type, and Macintosh operating system version on anatomical volume and cortical thickness measurements. PloS one 2012;7(6):e38234.
7. Glatard T, Lewis LB, Ferreira da Silva R, Adalat R, Beck N, Lepage C, et al. Reproducibility of neuroimaging analyses across operating systems. Frontiers in neuroinformatics 2015;9:12.
8. Gummaraju J, Desikan T, Turner Y. Over 30% of official images in DockerHub contain high priority security vulnerabilities. In: Technical Report; 2015.
9. Shu R, Gu X, Enck W. A Study of Security Vulnerabilities on DockerHub. In: Proceedings of the Seventh ACM on Conference on Data and Application Security and Privacy CODASPY '17, New York, NY, USA; 2017. p. 269–280.
10. Zerouali A, Mens T, Robles G, Gonzalez-Barahona JM. On the relation between outdated Docker containers, severity vulnerabilities, and bugs. In: 2019 IEEE 26th International Conference on Software Analysis, Evolution and Reengineering (SANER) IEEE; 2019. p. 491–501.
11. Gorgolewski KJ, Alfaro-Almagro F, Auer T, Bellec P, Capotă M, Chakravarty MM, et al. BIDS apps: Improving ease of use, accessibility, and reproducibility of neuroimaging data analysis methods. PLoS computational biology 2017;13(3):e1005209.
12. Glatard T, Kiar G, Aumentado-Armstrong T, Beck N, Bellec P, Bernard R, et al. Boutiques: a flexible framework to integrate command-line applications in computing platforms. GigaScience 2018;7(5):giy016.
13. Rampin R, Chirigati F, Shasha D, Freire J, Steeves V. ReproZip: The Reproducibility Packer. Journal of Open Source Software 2016;1(8):107.
