# Supplementary material for: An analysis of security vulnerabilities in container images for scientific data analysis
Source: Gigascience. 2021 Jun 3;10(6):giab025. doi: 10.1093/gigascience/giab025 (PMC8173661; doi:10.1093/gigascience/giab025)
Supplement: giab025_GIGA-D-20-00311_Revision_1 [file giab025_giga-d-20-00311_revision_1.pdf]

GigaScience

An Analysis of Security Vulnerabilities in Container Images for Scientific Data Analysis

--Manuscript Draft--

**Manuscript Number:**	GIGA-D-20-00311R1
**Full Title:**	An Analysis of Security Vulnerabilities in Container Images for Scientific Data Analysis
**Article Type:**	Technical Note
**Funding Information:**	Canada Research Chairs — Dr Tristan Glatard<br>Fonds de Recherche du Québec - Nature et Technologies — Dr Tristan Glatard
**Abstract:**	Software containers greatly facilitate the deployment and reproducibility of scientific data analyses in various platforms. However, container images often contain outdated or unnecessary software packages, which increases the number of security vulnerabilities in the images, widens the attack surface in the container host, and creates substantial security risks for computing infrastructures at large. This paper presents a vulnerability analysis of container images for scientific data analysis. We compare results obtained with four vulnerability scanners, focusing on the use case of neuroscience data analysis, and quantifying the effect of image update and minification on the number of vulnerabilities. We find that container images used for neuroscience data analysis contain hundreds of vulnerabilities, that software updates remove about two thirds of these vulnerabilities, and that removing unused packages is also effective. We conclude with recommendations on how to build container images with a reduced amount of vulnerabilities.
**Corresponding Author:**	Tristan Glatard<br>Concordia University<br>Montreal, CANADA
**Corresponding Author Secondary Information:**	
**Corresponding Author's Institution:**	Concordia University
**Corresponding Author's Secondary Institution:**	
**First Author:**	Bhupinder Kaur
**First Author Secondary Information:**	
**Order of Authors:**	Bhupinder Kaur
	Mathieu Dugré
	Aiman Hanna
	Tristan Glatard
**Order of Authors Secondary Information:**	
**Response to Reviewers:**	See letter and detailed answer included as attachment.
**Additional Information:**	
**Question**	**Response**
Are you submitting this manuscript to a special series or article collection?	No
**Experimental design and statistics**<br><br>Full details of the experimental design and statistical methods used should be given	Yes

Powered by Editorial Manager® and ProduXion Manager® from Aries Systems Corporation

in the Methods section, as detailed in our Minimum Standards Reporting Checklist. Information essential to interpreting the data presented should be made available in the figure legends.<br><br>Have you included all the information requested in your manuscript?	
**Resources**<br><br>A description of all resources used, including antibodies, cell lines, animals and software tools, with enough information to allow them to be uniquely identified, should be included in the Methods section. Authors are strongly encouraged to cite Research Resource Identifiers (RRIDs) for antibodies, model organisms and tools, where possible.<br><br>Have you included the information requested as detailed in our Minimum Standards Reporting Checklist?	Yes
**Availability of data and materials**<br><br>All datasets and code on which the conclusions of the paper rely must be either included in your submission or deposited in publicly available repositories (where available and ethically appropriate), referencing such data using a unique identifier in the references and in the "Availability of Data and Materials" section of your manuscript.<br><br>Have you have met the above requirement as detailed in our Minimum Standards Reporting Checklist?	Yes

Manuscript

Click here to access/download;Manuscript;Answer to the reviewers.pdf

UNIVERSITÉ Concordia UNIVERSITY

GINA CODY SCHOOL OF ENGINEERING AND COMPUTER SCIENCE

Department of Computer Science and Software Engineering

Montreal,
February 28th, 2021

Dear Editor and Reviewers,

We would like to thank you for considering our manuscript for publication in GigaScience and for the thorough reviews. Please find below our answer to your comments. The corresponding revision of our manuscript is attached, where revisions are highlighted in green.

We thank you again for your time and consideration, and we remain available for any further information you may need.

Sincerely,

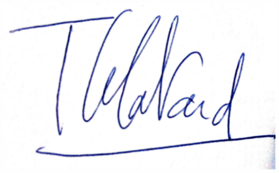

Tristan Glatard, PhD
Associate Professor, Department of Computer Science and Software Engineering, Concordia University
Canada Research Chair (Tier II) on Big Data Infrastructures for Neuroinformatics

1455 De Maisonneuve Blvd. W., EV 3.139, Montreal, Quebec, Canada H3G 1M8
Tel. 514-848-2424 ext. 3000 Fax 514-848-2830 cse.concordia.ca

Reviewer #1

This very interesting Technical Note reports on security vulnerabilities in software container images used for neuroscience data analysis. The authors report on two software container images - Docker and Singularity - and four software container image scanners - Anchore, Vuls, Clair, and Stools. In their analysis, the authors highlight an average of 460 vulnerabilities per software container image. As a means of reducing the number of vulnerabilities, the authors explored the effects of package update and image minification. The authors highlight that updating container images is a major factor in reducing the number of vulnerabilities, and in support of this the authors report that no vulnerabilities were to be found in base Docker images ubuntu:20.04 and centos:7 after package update. The authors additionally report that removing unused packages by image minification is also effective. An important point that the authors demonstrate is that both image minification and package update techniques are complementary methods for reducing the number of security vulnerabilities.

This analysis enabled the authors to provide a set of image creation guidelines on how to deliver a more secure deployment of image containers on HPC clusters. These guidelines suggest minifying container images by using lightweight OS distributions, and applying regular security updates. These are very useful guidelines for the neuroscience data management community. The manuscript is well written, and the scripts used for container vulnerability analysis are publicly available on GitHub and have been ascribed an OSI-approved GPL3 license. The authors additionally provide a Jupyter notebook that can be used to regenerate the figures shown in the manuscript.

I recommend this Technical Note for publication in GigaScience.

We thank the reviewer for the thorough review of our paper and the positive comments.

Reviewer #2

This Technical Note manuscript makes several interesting investigations into the security of containers for neuroimaging processing, and the topic is suitable for Gigascience. The authors analyze a number of common, publicly available containerized images from widely used processing pipelines. The analysis of security vulnerabilities is extensive and results in a number of concrete pieces of advice and best practices for software developers in the neuroimaging space. There are also implications for other fields which commonly use containerized pipelines.

I have several suggestions for improvements of this manuscript, which I hope will improve the impact to the community.

While it is addressed at various points in the manuscript, the key issue within the neuroimaging community with regards to updating images and packages is the fundamental tension between reproducibility (by pinning the exact package versions and OS used) and security through using updated packages. The point needs to be made more clearly that security vulnerabilities need to be taken more seriously while providing more insight into the issues that can arise when updating packages. Some possible suggestions to address this:

1. In the introduction, you list several key questions. I think an additional question, or perhaps a modification to question two, is how much security vulnerabilities can be reduced without introducing errors or inconsistencies in processing data?

This is a very relevant point indeed. However, the question of results stability with respect to security updates is a difficult one that we believe would require a study on its own. Software updates induce changes in data processing mainly through numerical perturbations that propagate in the analyses. We believe that the effect of software updates should be assessed by evaluating the numerical stability of analyses and correct them if necessary, as explained in the initial discussion ("we advocate a more systematic analysis of the numerical schemes involved in data analyses, which, coupled with software testing, would make the analyses robust to software updates."). To take into account the reviewer's comment, we rephrased in the introduction the description of the factors leading to outdated software.

2. In the results, can you expand on the potential impact of the security vulnerabilities found? You highlight a few serious vulnerabilities, but have one sentence on the potential issues. Are there additional ramifications which would motivate the neuroimaging software developer to update? Perhaps jeopardizing data or user credentials to access online systems? This may allow you to be more specific in the last two paragraphs of the discussion as well in terms of actual security vulnerabilities found in these containers.

We revised the results paragraph on detected vulnerabilities as follows to answer the reviewer's comment:

> *Remote attackers could possibly exploit these vulnerabilities to execute arbitrary code in the container or to store arbitrary files in the system. For instance, vulnerability CVE-2019-3462 could lead to remote code execution through human-in-the-middle attacks and vulnerability CVE-2018-1000802 could lead to arbitrary file injections in the filesystem through unfiltered Python user input. Among other consequences, remote code execution and injection of arbitrary files could be leveraged to steal user credentials, to use CPU cycles or storage space for illegitimate purposes, or to attempt denial-of-service attacks against the system.*

We also mentioned the possible stealing of user credentials and arbitrary code or file injection in the last two paragraphs of the discussion.

3. In the results, in the "Effect of image update" you mention "in spite of the associated reproducibility challenges"- did you run into any issues in these updates you can specifically report? This may provide insight into the process of upgrading neuroimaging containers.

Updating the container images did not break the tested analyses. For the reasons mentioned previously, we did not investigate possible consequences on numerical stability. We added two sentences to section "Effect of image update" to reflect the reviewer's comment.

4. In the results, in the "Effect of minification" you mention "It is a tedious operation, as it requires running an actual data analysis in the container image". I think this is also worth expanding on. Were there test examples, unit tests, or integration tests from BIDS or Boutiques used for this?

The Boutiques specification indeed allows developers to specify analysis tests, but we found out that this feature wasn't consistently used in the images. Therefore we relied on analysis examples found in the documentation of the applications. We explained this in the "Image minification" section of the methods.

Did the minification tools create any errors in these images? Was there any insight in which kinds of packages were removed?

Minification did not create any errors in these images as all the dependencies were taken care of. Common packages removed by the minification process were compilers, unused Python packages, and unused file compression utilities. We mentioned this in section "Effect of minification".

Were there commonalities in the packages which had security issues which could not be removed from minification?

No particular commonality could be found in these packages.

Addressing these points would help the neuroscientist and neuroimaging software developer understand the risks associated with outdated containers as well as the path to upgrade.

Some additional minor comments and questions

1. Were there significant differences between the kinds of errors found in docker and singularity images, or the potential severity of those errors given the improved user privilege control of singularity?

There were no significant differences in the vulnerabilities found in Docker or Singularity. Scanners detect vulnerabilities through the package manager and do not involve the container engine in this process.

2. Does the docker engine or singularity engine version matter? Does the use of an orchestration system like docker compose matter? I am not sure but I am curious.

Vulnerabilities exist in container images independently from the container execution context. Container engines and orchestration systems would certainly play a role in the attacks that might exploit the detected vulnerabilities, but they do not interfere with the detection of vulnerabilities itself.

Even more minor comments:

1. Bigger text on Figure 1 would be helpful.

We increased the font size by 10%.

Reviewer #3

Review summary

This paper presents a little study of selected set of popular containerized applications (BIDS-Apps, Boutiques) regarding the number of known vulnerabilities found to be present in the contained within containers software components. Two approaches to reduce number of known/detected vulnerabilities were approached: container updates and/or minimization. Overall it is a nice and informative paper. I only have once notable concern: recommendation "ii. Use lightweight base images such as Alpine Linux" . As described in greater detail below, I think at large it would only potentially only "hide" the problem away by making vulnerabilities undetected (but not "not present").

Thank you for the thorough review of our paper and the relevant comments. As detailed below we agree with your comments on the use of lightweight base images and updated the manuscript accordingly.

Abstract

** ", [especially] on high-performance computing clusters (HPC)".

Since the statement is IMHO applicable in general, not only to HPC.

Similar statement in Introduction opening.

I could even argue that taking "possession" over a local desktop could provide an attack vector enabling intruder to access multiple HPC systems a user might have access to. So I would not "limit" to HPC alone, but rather add a statement that such vulnerabilities might affect HPC deployments at a larger scale, while circumventing/obliterating security measures system administrators put in place.

We agree with you completely. In the abstract, introduction, discussion and conclusion we replaced the mention of containers "deployed on HPC clusters" by "used in scientific data analyses". We added a paragraph to the introduction explaining how scientific data analyses involve a large range of systems that could all be attacked should a single one of them be compromised.

Introduction

* "and [often can] control the memory, CPU, network and file-system resources"

Since AFAIK Singularity by default would not bother to control memory/CPU/network or even file resources. That was the point behind it: to be (unlike Docker) very transparent to the process and as close to the "chroot" lightweight as possible.

Fixed.

* "for Docker and appc container"

Maybe that originally used version promoted support for appc, but as appc specification was stopped to be developed in favor of OCI, Clair now states support for OCI (not appc). So it might be better to say "Docker and OCI container images".

Fixed.

* "give scanning results" -> "list scanning results"

Minor, and I do not like "list" either but have not come up with a better alternative

Replaced with "report scanning results".

* "In comparison, no vulnerabilities were found in base Docker images ubuntu:20.04 and centos:7 after package update. "

That it IMHO would be inappropriate comparison to make: non-updated applications images to updated stock images. Why not to also provide a number of vulnerabilities in base images **BEFORE** the update? That would also deliver the point that even base images could have vulnerabilities. If no longer possible to do easily (no access to the original base images), just remove "In comparison,"?

We removed the comparison.

* Figure 1 -- I cannot tell between "critical" and Ubuntu -- both are vivid red

Since it has a (n) anyways, why just to make it that solid color which is for High ATM and adjust all the rest accordingly? would make figure more consistent IMHO

Critical and high vulnerabilities are now represented by the same solid color (alpha=1). The number of critical vulnerabilities is still represented in brackets. Unknown statuses are now represented in white

(alpha=0) to further reduce the number of transparency levels used in the figure. The caption of Figure 1.A was also updated to clarify the intent of the legend.

* "six of them could not be updated due to various issues with the package manager,"

Too vague. Most likely it was not an "issue" per se but a. base distribution is EOLed and APT lines had to be adjusted (was not done), or key expired (also could be worked around). I would have advised to rephrase with a bit more clearer statement on why they were not updated - as "update" is promoted as an effective way to address vulnerabilities, inability (or difficulty) to update is an important factor!

We added text to report on the specific issues encountered while updating these images.

* Is minimization "useful" to address security issues?

Please state and support your opinion, since IMHO minimization is of no direct effect since vulnerable minimized-away software packages (even though shipped within container) are not involved in the computation anyways. Describe how/when they could potentially be harmful (e.g. a user unintentionally triggers those packages execution, which should be unlikely if container user through itscomputational entry point).

The coin could also be flipped: only a port of vulnerabilities would in the containers could be relevant to the computational workflow. Judging from Fig 1B it actually could be 25-100% (I suspect g was minified by its developers)

Vulnerabilities present in unused packages increase the risk of escalation attacks after an attacker exploited a vulnerability directly involved in the computation. Here is a possible scenario:

- Attacker submits containerized Python 2.7 jobs through a web portal such as OpenNeuro or CBRAIN. CVE-2018-1000802, which can be exploited for file injection, was fixed in Python 2.7 interpreter used in the analysis but the container image contains an unused older version of Python 2.7 that has the vulnerability.
- Attacker exploits a shortcoming in the web portal to change the PATH environment variable in the container and make it point to the older Python interpreter that has CVE-2018-1000802.
- Attacker exploits CVE-2018-1000802 to inject malicious files in the container.

We added a sentence at the beginning of section "Image minification" to clearly state our opinion that vulnerabilities in unused packages should be avoided to reduce the risk of escalation attacks.

Discussion

** i. Introduce software dependencies cautiously

agree. you could provide immediate hints such as `--no-install-recommends` for `apt install` invocations.

We added a comment on this in paragraph (i).

For this and the rest of the recommendations, I think readers would greatly benefit from addition of more specific references and examples, e.g. for "vi. Run image scanners during continuous integration" -- is there a project/container you could refer to as an example?

We added references on how to use CI with Anchore and Stools.

** ii. Use lightweight base images such as Alpine Linux.

disagree, since it could also fire back. I hypothesize: The fact that you found less vulnerabilities on alpine-based images could be due to the fact that dependencies were manually built/installed (or some other distribution like conda was used) and thus such "custom" installations simply were not scanned by vulnerability scanners. "update" of such containers becomes infeasible. As a result you just end up just amplifying the problem: vulnerabilities cannot be detected (but exist), updates are not possible. Yes, such images would be "smaller", which will be good, but you are hiding the elephant with such suggestion.

Thanks for this excellent point. We completely agree that installing dependencies by circumventing the package manager in such distributions would only worsen the problem. We replaced this recommendation by a note on lightweight distributions in point (i).

Similarly a statement in the conclusions should be adjusted, which ATM just recommends "using lightweight OS distributions".

This statement was rephrased. We also removed the related comment about Alpine in section "Detected vulnerabilities".

** iii. Use OS releases with long-term support.

You should note that "base OS" LTS or not support relates to only packages provided through the distribution. It provides no magical means for vulnerabilities fixup for scientific software installed manually or from additional repositories (like NeuroDebian, NeuroFedora, conda-forge, etc). BUT if scientific software components are built "properly", dynamically linking against distribution provided libraries, then updates of the base distribution would automagically address vulnerabilities within scientific components (which would not happen if they are either built statically embedding all the libraries, or just bundling them for distribution -- e.g. like standalone distributions of FSL, FreeSurfer etc would do).

So, by itself, stable base is not a guarantee that container would be "safer" after update. Scientific components should ideally be integrated within the distribution itself. The follow up "iv. Install packages, not files" is a good one, but IMHO text here should be a bit more explicit on aforementioned aspect. It could also "link" into that aspect of "could not be updated due to various issues with the package manager," -- how many of those were not based on LTS of some kind?

The recommendation to "install packages, not files" was rewritten to take this comment into account. The new version mentions the limited inclusion of scientific software in base OS images, the suggestion to dynamically link third-party software to base OS libraries, and precise the added-value of domain specific repositories.

*** "and Debian stable releases are maintained for 3 years"

There is Debian LTS support, after "stable release support", which is provided for at least 5 years: https://wiki.debian.org/LTS

The text was fixed accordingly.

** "v. Minify container images. "

and explicitly 'link' to i. on cautious introduction of dependencies? might even better be reordered so those two advises come close.

This recommendation is now mentioned after (i) and explicitly refers to it.

Manuscript Click here to access/download;Manuscript;paper.pdf

Placeholder for OUP logo oup.pdf

(GIGA)^n SCIENCE

GigaScience, 20xx, 1–6

doi: xx.xxxx/xxxx
Manuscript in Preparation

An Analysis of Security Vulnerabilities in Container Images for Scientific Data Analysis

Bhupinder Kaur[1], Mathieu Dugré[1], Aiman Hanna[1] and Tristan Glatard[1]

[1]Department of Computer Science and Software Engineering, Concordia University, Montreal, Canada

Abstract

Software containers greatly facilitate the deployment and reproducibility of scientific data analyses in various platforms. However, container images often contain outdated or unnecessary software packages, which increases the number of security vulnerabilities in the images, widens the attack surface in the container host, and creates substantial security risks for computing infrastructures at large. This paper presents a vulnerability analysis of container images for scientific data analysis. We compare results obtained with four vulnerability scanners, focusing on the use case of neuroscience data analysis, and quantifying the effect of image update and minification on the number of vulnerabilities. We find that container images used for neuroscience data analysis contain hundreds of vulnerabilities, that software updates remove about two thirds of these vulnerabilities, and that removing unused packages is also effective. We conclude with recommendations on how to build container images with a reduced amount of vulnerabilities.

Key words: Containers; Docker; Singularity; Security Vulnerabilities; Neuroimaging.

Introduction

Software containers have emerged has an efficient solution to deploy scientific data analyses in various platforms, due to their portability, ease of use, and limited overhead. Taking advantage of core Linux kernel features such as namespaces, control groups, and chroot, containers isolate processes from the host computer and often can control the memory, CPU, network, and file-system resources assigned to them. However, containers still share the kernel, mounted file systems and some devices with the host, which raises security concerns [2, 3, 4] and opens the door to privilege escalation, denial of service, information leak, and other types of attacks [5].

Container images typically include full operating system (OS) distributions in addition to data analysis software and their dependencies. They are rarely updated due to concerns that updated software may be incompatible or may interfere with the results via numerical perturbations propagating in the analyses in unknown ways [6, 7]. Images also typically include more dependencies than required, to make them easier to reuse between experiments. As a result, over 30% of official images in DockerHub have been shown to contain high-priority security vulnerabilities [8], images on average contain over 180 vulnerabilities [9], and vulnerabilities are often caused by outdated packages [10].

Scientific data analyses typically involve a range of computational infrastructures, including personal workstations, lab servers, HPC clusters, and cloud computing platforms. It is common for researchers to have access to multiple systems through a combination of credentials and to migrate analyses depending on their evolution. As a result, an attacker gaining access to one of these systems, possibly through a vulnerable container, might be able to compromise an extensive infrastructure and use it for malicious purposes.

In this study, we focus on the vulnerabilities present in container images used in scientific data analysis, in particular in the neuroimaging domain. We address the following questions:

What is the current amount of vulnerabilities in container images used in scientific analyses? Vulnerabilities are possible attack vectors that can seriously compromise the security of computing systems and the integrity of user data. We report vulnerability scans produced by four popular image scanning tools: Anchore, Vuls, Clair, and Singularity tools.

Can the amount of vulnerabilities be reduced by updating the images? To avoid breaking software dependencies and introducing numerical perturbations, container images used in scientific analyses often include outdated software. We report on the effect of software updates on the amount of vulnerabilities found in images.

Can the amount of vulnerabilities be reduced by minifying images? Container images often include more software packages than necessary for a typical analysis. We report on the impact of unused software packages on the presence of vulnerabilities.

The remainder of this paper describes the container images and scanners used in our experiment, and our methodology for updating and minifying images. Results present the vulnerabilities detected in container images, quantify the effectiveness of updating and minifying images, and explain the differences observed between scanners. In conclusion, we provide a set of image creation guidelines for a more secure deployment of containers in scientific analyses.

OS	Anchore	Vuls	Clair
**Alpine**	Alpine-SecDB	Alpine-SecDB	Alpine-SecDB
**CentOS**	Red Hat OVAL Database	Red Hat OVAL Database and Red Hat Security Advisories	Red Hat Security Data
**Debian**	Debian Security Bug Tracker	Debian OVAL Database and Debian Security Bug Tracker	Debian Security Bug Tracker
**Ubuntu**	Ubuntu CVE Tracker	Ubuntu OVAL Database	Ubuntu CVE Tracker

Table 1. Vulnerability databases used by scanners for different OS distributions. All scanners also refer to the National Vulnerability Database (NVD) for vulnerability metadata.

Materials and Tools

We used container images from two popular application frameworks, as well as four of the major image scanners.

Container Images

We scanned all container images available at the time of this study on two containerization frameworks used in neuroscience: BIDS apps [11] (26 images) and Boutiques [12] (18 images), totalling 44 container images. At the time of the study, BIDS apps had 27 images, out of which one wasn't available on DockerHub. Boutiques had 49 images, however, only 23 unique images were listed, out of which 3 couldn't be retrieved and 2 were already included in BIDS apps. All the final 26 images from BIDS apps were Docker images, whereas the 18 Boutiques images contained 12 Docker images and 6 Singularity images.

Image Scanners

We compared the results obtained with four container image scanners: Anchore, Vuls, and Clair to scan Docker images, and Singularity Container Tools (Stools) to scan Singularity images.

Anchore is an end-to-end, open-source container security platform. It analyzes container images and lists vulnerable OS packages, non-OS packages (Python, Java, Gem, and npm), and files. In our experiments, we used Anchore Engine version 0.5.0 through Docker image `anchore/anchore-engine:v0.5.0`, and Anchore vulnerability database version 0.0.11.

Vuls is an open-source vulnerability scanner for Linux and FreeBSD. It offers both static and dynamic scanning, and both local and remote scanning. In our experiments, we used Vuls 0.9.0, executed through Docker image `vuls/vuls:0.9.0` in remote dynamic mode.

Clair is an open-source and extensible vulnerability scanner for Docker and OCI (Open Container Initiative) container images, developed by CoreOS (now Container Linux), a Linux distribution to deploy container clusters. We used Clair through Clair-scanner, a tool to facilitate the testing of container images against a local Clair server. We used Clair version 2.0.6, executed through Docker image `arminc/clair-local-scan:v2.0.6`. For the vulnerability database, we used Docker image `arminc/clair-db:latest`, last updated on 2019-09-18.

Singularity Tools (Stools) are an extension of Clair for Singularity images. Stools exports Singularity images to `tar.gz` format, acting as a single layer Docker image to circumvent the Docker-specific requirements in the Clair API. In our experiments, we used Singularity Tools version 3.2.1 through Docker image `vanessa/stools-clair:v3.2.1`. Since Stools uses Clair internally for scanning, the vulnerability databases used by Stools are the same as mentioned for Clair. To scan Singularity images, we followed the steps mentioned in the Stools documentation.

Vulnerability Databases

Scanners refer to two types of vulnerability databases (Table 1). The first one is the Open Vulnerability and Assessment Language (OVAL) database, an international open standard that supports various OS distributions including Ubuntu, Debian and CentOS but not Alpine. The second one are vulnerability databases from specific OS distributions, such as Alpine-SecDB, Debian Security Bug Tracker, Ubuntu CVE Tracker, or Red Hat Security Data. In these databases, OS distributions often assign a status to each vulnerability, to keep track of required and available security fixes in different versions of the distribution. Vuls uses OVAL databases for all distributions except Alpine. On the contrary, Clair exclusively refers to distribution-specific databases. Anchore uses OVAL only for CentOS, as distribution-specific databases are assumed to be more complete. It is also worth noting that there is no vulnerability data for Ubuntu 17.04 and 17.10 distributions in the OVAL database, since these distributions have reached end of life, meaning that images with these distributions cannot be scanned with Vuls.

For CentOS images, Anchore and Clair report scanning results using Red Hat Security Advisory (RHSA) identifiers, whereas Vuls uses the Common Vulnerabilities and Exposures (CVE) identifiers used in OVAL. We mapped RHSA identifiers to corresponding CVE identifiers, to allow for a comparison between scanners.

Different vulnerabilities may be reported by scanners if scanning experiments take place on different dates. To avoid such discrepancies, we froze the vulnerability databases used by these scanners as of 2019-09-25.

Image Update

A first approach to reduce the number of vulnerabilities in container images is to update their packages to the latest version available in the OS distribution. To study the effect of such updates, we created a script (available here) to identify the package manager in the image, and invoke it to update all OS packages. We updated images on 2019-11-05.

Image Minification

A second approach to reduce the number of vulnerabilities in the images is to remove unnecessary packages, an operation potentially specific to each analysis. Although vulnerabilities in unused packages could not be directly exploited when running the analysis provided by the container image, they still increase the risk of escalation attacks and should therefore be avoided.

We used the open-source ReproZip tool [13] to capture the list of packages used by an analysis. ReproZip first captures the list of files involved in the analysis, through system call interception, then retrieves the list of associated software packages, by querying the package manager. We extend this list with a passlist of packages required for the system to function, such as `coreutils` and `bash`, and with all the dependencies of the required packages, retrieved using Debtree. Repoquery could be used in RPM-based distributions instead. Our minification script, available here, installs ReproZip in the image to minify, runs an analysis to collect a ReproZip trace, and finally deletes all unnecessary packages. We had used the Neurodocker tool initially, but it did not affect the detected vulnerabilities as it was removing unused files without using the package manager.

Image minification is a tedious operation, as it requires (1) creating relevant analysis examples and (2) running these examples in the container image, to identify the packages required by the ap-

plication. The resulting minified container image is only valid for the examples selected in the minification process, as other executions might require a different set of packages.

While the Boutiques specification allows developers to specify analysis tests, we found that this feature was not consistently used in the studied container images. Therefore, we relied on analysis examples found in the documentation of the applications. Using this approach, we minified five Debian- or Ubuntu-based BIDS app images.

Results

Figure 1 presents our results. All the collected data are available in our GitHub repository at https://github.com/big-data-lab-team/container-vulnerabilities-paper with a Jupyter notebook to regenerate the figures.

Detected Vulnerabilities

An important amount of vulnerabilities were found in the tested container images (Fig 1-**A**), with an average of 460 vulnerabilities per image and a median of 321. Moreover, a significant fraction of detected vulnerabilities are of high severity (CVSS score >=7.0) and a few of them are of critical severity (CVSS >= 9.0). Remote attackers could possibly exploit these vulnerabilities to execute arbitrary code in the container or to store arbitrary files in the system. For instance, vulnerability CVE-2019-3462 could lead to remote code execution through human-in-the-middle attacks and vulnerability CVE-2018-1000802 could lead to arbitrary file injections in the filesystem through unfiltered Python user input. Among other consequences, remote code execution and injection of arbitrary files could be leveraged to steal user credentials, to use CPU cycles or storage space for illegitimate purposes, or to attempt denial-of-service attacks against the system.

Images based on the Alpine distribution had the lowest numbers of vulnerabilities, but no significant difference in the numbers of vulnerabilities detected in Ubuntu, Debian or CentOS distributions was observed.

Unsurprisingly, a strong linear relationship is found between the number of detected vulnerabilities and the number of packages present in the image (Fig 1-**C**, r=0.82, $p < 10^{-11}$). On average, 1.7 vulnerabilities are introduced for each new package installation. This observation motivates a systematic review of software dependencies by application developers, to avoid unnecessary packages in container images. Compared to Ubuntu and Debian distributions, CentOS images seem to have a lower number of vulnerabilities by package on average, although data is too scarce to conclude.

Effect of image update

Updating container images reduces the number of vulnerabilities by package by a factor of 3 on average, resulting in only 0.6 extra vulnerabilities by package (Fig 1-**D**, r=0.81, $p < 10^{-7}$). Twelve container images are missing on this figure. Six of them are Singularity images that we did not update and six of them could not be updated by our script due to the following issues. One image was built from base Docker image CentOS 7.1.1503 that includes package `fakesystemd` conflicting with several other distribution packages: updating would require either updating the base image or swapping package `fakesystemd` for `systemd`. Three images were build from Ubuntu 17.04 or Debian 8 that reached end of life: updading would require changing the source list to make it point to old releases. Two images could not be updated because some files previously installed through the package manager were removed by other means, leading to failure of the update process. Software updates did not break the tested analyses in the remaining images. We did not investigate the potential consequences of image updates on numerical stability as it would require a full separate study. Updating packages appears to be an efficient way to avoid vulnerabilities. It is not an ultimate solution though, as a substantial number of vulnerabilities remain.

Effect of minification

Using the ReproZip-based approach described previously, we minified 5 different images covering the spectrum of detected vulnerabilities (Fig 1-**B**). We find that minification reduces the number of vulnerabilities, albeit less systematically than package update. For some container images, such as image **S**, minification removes more than 70% of the detected vulnerabilities. For other images, such as image **g**, it only reduces the number of vulnerabilities by less than 1%. The effect of minification stems from the number of packages that can be removed, which varies greatly across images. For instance, images **g** and **a** have a large number of packages, but the last majority of them is required by the analysis, which makes minification less useful. In other cases, a limited number of unnecessary packages contain a significant number of vulnerabilities, which makes minification very impactful. This was the case in images **d**, **S** and **U**, where removing compilers and kernel headers reduced the number of vulnerabilities by an important fraction. Minification did not create any errors in these images as all the dependencies were taken care of properly. Common packages removed by the minification process were compilers, unused Python packages, and unused file compression utilities.

Combined effect of image update and minification

Package update and image minification remove different types of vulnerabilities. The former is efficient against vulnerabilities that have been fixed by package maintainers, while the latter targets unused software. In two of the five tested images (images **S** and **U**), we find that combining update and minification further reduces the number of vulnerabilities compared to using only one of these processes (Fig 1-**B**).

Differences between scanners

The results presented so far were obtained with Anchore (Docker images) and Stools (Singularity images). We scanned the Docker images with two other tools, Clair and Vuls, to evaluate the stability of our results. Important discrepancies were found between scanners (Fig 2), in particular between Anchore and the other two scanners, for which Jaccard coefficients as low as 0.6 were found, meaning that scanning results only overlapped by 60%. Vuls and Clair appear to be in better agreement, with a Jaccard coefficient of 0.8.

We analyzed these results and explained some reasons behind the observed discrepancies. Out of 4453 vulnerabilities detected by Anchore only (region **1** in Fig 2), 4443 are found in the development package of the C library (`linux-libc-dev` in Ubuntu and Debian). Clair detects only Debian vulnerabilities in `linux-libc-dev`, whereas Vuls do not detect vulnerabilities in this package at all. Since Anchore ignores Debian vulnerabilities flagged as `minor`, it might either detect (region **2**) or ignore (region **3**) the Debian vulnerabilities detected by Clair in `linux-libc-dev`. The remaining 10 vulnerabilities in region **1** are found in sub-packages of vulnerable packages: they are correctly reported by Anchore and missed by Vuls and Clair.

Many vulnerabilities in region **3** and **4** are from images based on Ubuntu 14.04. In the Ubuntu CVE tracker database used by Clair and Anchore, there are two entries for Ubuntu 14.04: one for LTS (Long-Term Support), a Ubuntu release with 5 years of techni-

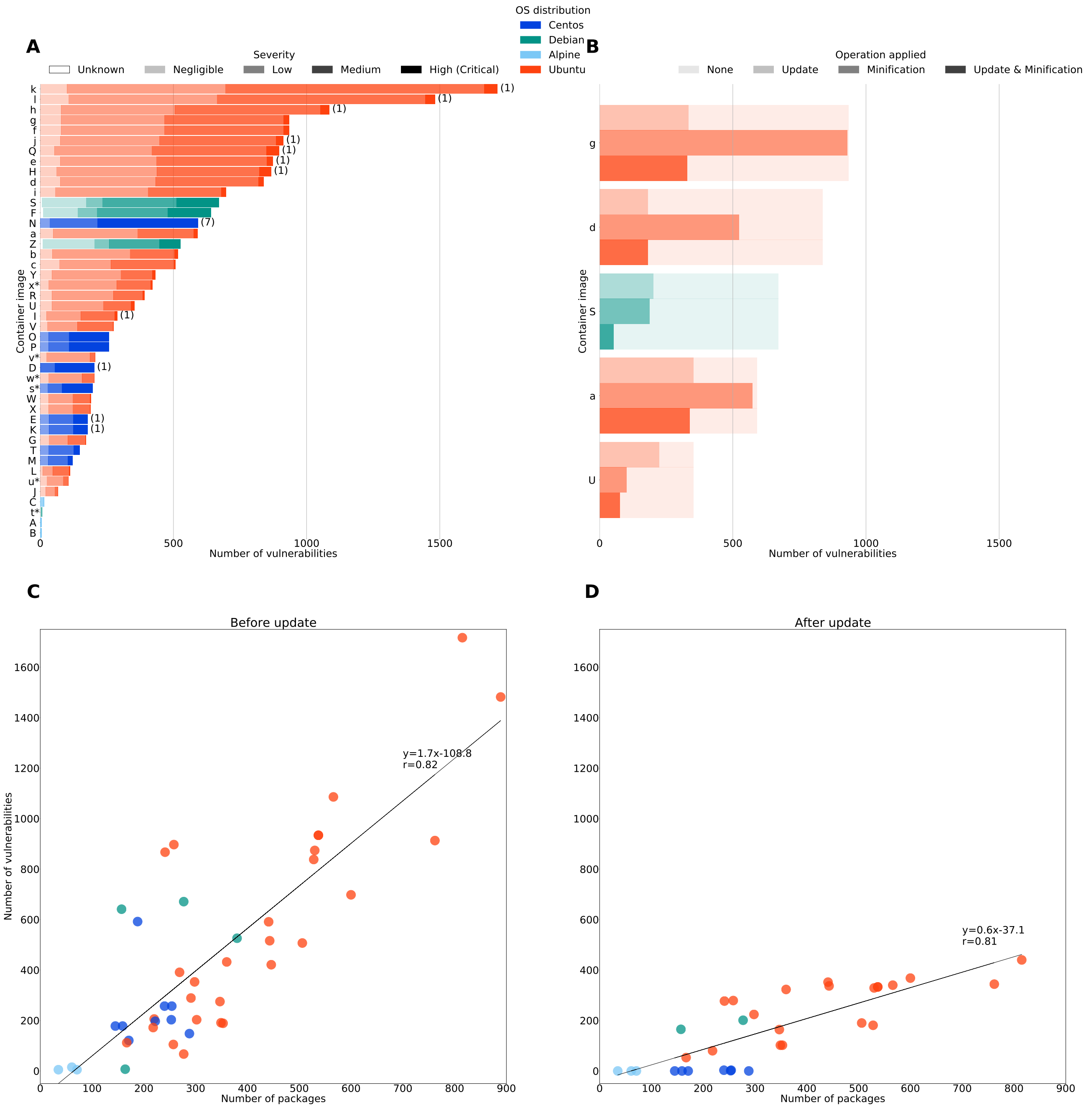

Figure 1. Number of vulnerabilities detected by Anchore and Stools in container images. **(A)** Number of vulnerabilities by container image and severity, showing hundreds of detected vulnerabilities per image. Images s*,t*,u*,v*,w* and x* are Singularity images scanned by Stools and others are Docker images scanned using Anchore. High and critical vulnerabilities are represented with the same solid color and critical vulnerabilities are reported between brackets. **(B)** Effect of image minification and package update on 5 container images, showing that both techniques are complementary **(C)** Number of vulnerabilities by number of packages, showing a strong linear relationship. **(D)** Number of vulnerabilities by number of packages *after package update*, showing that software updates importantly reduce the number of detected vulnerabilities.

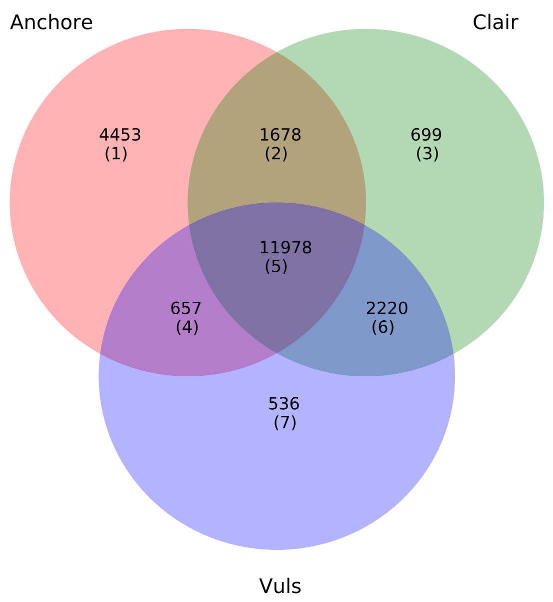

Figure 2. Differences between vulnerabilities detected by the different scanners. The Jaccard coefficients between the sets of detected vulnerabilities are quite low, showing important discrepancies between the scanners: Jaccard(Anchore, Clair) = 0.63, Jaccard(Anchore, Vuls) = 0.59, Jaccard(Vuls, Clair) = 0.80. Two Ubuntu 17.04 images weren't included in this comparison as they cannot be scanned by Vuls.

cal support, and another one for ESM (Extended Security Maintenance), a release that provides security patches beyond the 5 years covered by LTS. Although all the scanned images are LTS, Clair refers to the ESM database entry while Anchore and Vuls refer to the LTS database entry. The vulnerabilities present in region **3** due to this discrepancy are incorrectly missed by Anchore and Vuls: they have been detected in ESM but were already present in LTS. The vulnerabilities in region **4** are incorrectly missed by Clair: they have been fixed in ESM but are still present in LTS.

Some vulnerabilities in region **6** are due to bugs in Anchore: the *epoch bug* ignores vulnerabilities related to package versions that contain an epoch (`:`); the *out of standard bug* ignores vulnerabilities that are ignored by the Ubuntu distribution. We reported these bugs to the Anchore developers through their Slack channel. Some vulnerabilities in region **6** are also due to the fact that Anchore intentionally ignores Debian vulnerabilities flagged as `minor`.

Finally, 32 vulnerabilities that are flagged temporary by the Debian distribution are reported by Vuls but not by Anchore or Clair (region **7**). The remaining 504 vulnerabilities in this region are all found in CentOS images. We weren't able to explain why they were detected by Vuls only.

Discussion

There is a widespread issue with security vulnerabilities in container images used for neuroimaging analyses, and it is likely to impact other scientific disciplines as well. As shown in our results, it is common for container images to hold hundreds of vulnerabilities, including several of critical severity. Container images are impacted regardless of the type of analyses that they support, and the main OS distributions Ubuntu, Debian and CentOS are all affected.

Software updates remove about two-thirds of the vulnerabilities found and should certainly be considered the primary solution to this problem. However, in neuroimaging as in other disciplines, software updates are generally discouraged because they can affect analysis results by introducing numerical perturbations in the computations [6, 7]. We believe that this position is not viable from an IT security perspective, and that it could endanger the entire Big Data processing infrastructure. Instead, we advocate a more systematic analysis of the numerical schemes involved in data analyses, which, coupled with software testing, would make the analyses robust to software updates. As a first step, the packages impacting the analyses could be specifically identified and the others updated, which would largely remove vulnerabilities.

Ultimately, software updates should even occur at runtime rather than when the container image is built. Indeed, it is likely that container images used for scientific data analyses be built only occasionally, perhaps every few weeks when a release becomes available, which may not be compatible with the frequency of required security updates. In fact, there is no definite reason for the application software release cycle to be synchronized with security updates, and security updates shouldn't be dependent on application software developers. Instead, we think it would be relevant for analytics engines to (1) systematically apply security updates when containers start, and (2) run software tests provided by application developers, including numerical tests, before running analyses.

Implementing such a workflow, however, requires a long-term endeavour to evaluate broadly the stability of data analysis pipelines, and to develop the associated software tests. For the shorter term, we identified the following recommendations for application developers to reduce the number of security vulnerabilities in container images:

i. *Introduce software dependencies cautiously*. Software dependencies come with a potential security toll that is often neglected. For instance, it can be tempting to add a complete toolbox to implement a relatively minor operation in a data analysis pipeline, such as a data format conversion, while the same functionality might be available in the existing dependencies of the pipeline, albeit in a less convenient way. Some package managers such as `apt` also support "weak" package dependencies that are recommended but not necessarily required for installation. Installation of such suggested packages can be avoided using specific options of the package manager (`-no-install-recommends` in the case of `apt`). Using lightweight base OS images such as Alpine Linux can also reduce the number of unnecessary dependencies. However, developers should ensure that this doesn't lead to installing extra dependencies without using the package manager, as mentioned in point (iv) below.

ii. *Minify container images.* Minifying container images is another way to reduce software dependencies. However, the automated minification process that we used in our study is unwieldy for a routine use, as it requires capturing execution traces with ReproZip to reconstruct the graph of package dependencies required for the analysis. In practice, it would be more practical for software developers to identify and remove unnecessary dependencies when they build containers, based on their knowledge of the application.

iii. *Use OS releases with long-term support.* Security updates are not provided for OS distributions that reached end of life. When a given release of a data analysis pipeline is expected to be used over a long period of time, typically several years as it is common in neurosciences, the life cycle of the distribution release should be considered when choosing a base container image. OS distributions have very different life cycle durations, as long and short life cycles serve different purposes. For instance, among RedHat-based distributions, Fedora release a new version every 6 months and provide maintenance for about a year, while CentOS release every 3-5 years and provide maintenance for 10 years. Similarly, Ubuntu and Debian LTS (long-term support) distributions provide security updates for at least 5 years.

iv. *Install packages, not files*. Regardless of their support status, base OS distributions covered by the previous recommendation rarely include scientific software. Since vulnerability scanners detect vulnerabilities from the list of installed packages, vulnerabil-

ities contained in files installed without the package manger remain undetected. To reduce such occurrences, scientific software should as much as possible be linked dynamically against libraries provided by the base OS distribution. The use of domain-specific repositories such as NeuroDebian or NeuroFedora in neuroimaging is also useful in this respect, as these distributions facilitate transparent software updates and might be covered by image scanners in the future.

v. *Run image scanners during continuous integration.* Scanning container images can be a cumbersome process that could be asynchronously executed during continuous integration (CI), through tools such as Travis CI or Circle CI. Including security scans in CI also allows developers to identify vulnerabilities quickly, before new software versions are released. The Anchore documentation includes specific instructions on how to do so at `https://anchore.com/cicd`. The Stools repository also includes a Travis CI file that can be reused for this purpose.

Describing specific attacks that would exploit vulnerabilities in container images is out of the scope of our study. We believe that such attacks are likely to exist given that critical vulnerabilities allowing for arbitrary code execution or file injection were found. Attacking systems through containers remains challenging due to their relative isolation from the host system. Under the assumption that container host users can be trusted, attackers would have to be remote, either in the same network or on a remote network. Two main types of attacks can be envisaged in these conditions: network-based attacks, exploiting vulnerabilities in network clients installed in the container, and data-based attacks, exploiting vulnerabilities through the processing of malicious data injected through third-party systems.

Several types of escalation attacks could be envisaged once remote attackers gain access to the container, in particular related to (1) stealing user credentials, (2) using the resources allocated to the container for malicious use, resulting in denial of service, and (3) attacking a host network service, for instance a locally-accessible server or a file system daemon. Exploits in the host kernel to break out of the container are always possible but unlikely assuming that the host system is maintained by professional system administrators.

Conclusion

Most container images used in scientific data analyses contain hundreds of security vulnerabilities, many of which are critical. In the short term, application software developers can address this issue by: (1) reducing software dependencies, and (2) applying regular security updates, which requires using OS distributions with long-term support. Longer term, data analysis pipelines would benefit from in-depth stability analysis, to ensure that analytical results are not affected by security updates.

This conclusion is not an alarming message urging system administrators to ban containers from their systems. User-controlled container images are just one of many end-user artifacts that could serve as attack vectors, and to our knowledge no attack has been described to exploit them. More traditional types of attacks targeting user credentials or network connections are likely to remain more common.

Availability of Data and Materials

The data and scripts used in this study are available in the GitHub repository at `https://github.com/big-data-lab-team/container-vulnerabilities-paper` with a Jupyter notebook to regenerate the figures. This manuscript is based on version 0.2 of the repository, available as DOI 10.5281/zenodo.4569271.

Competing interests

The authors declare that they have no competing interests.

References

1. Kurtzer GM, Sochat V, Bauer MW. Singularity: Scientific containers for mobility of compute. PloS one 2017;12(5):e0177459.
2. Martin A, Raponi S, Combe T, Di Pietro R. Docker ecosystem–vulnerability analysis. Computer Communications 2018;122:30–43.
3. Sultan S, Ahmad I, Dimitriou T. Container Security: Issues, Challenges, and the Road Ahead. IEEE Access 2019;7:52976–52996.
4. Combe T, Martin A, Di Pietro R. To Docker or not to Docker: A security perspective. IEEE Cloud Computing 2016;3(5):54–62.
5. Gantikow H, Reich C, Knahl M, Clarke N. Providing security in container-based HPC runtime environments. In: International Conference on High Performance Computing; 2016. p. 685–695.
6. Gronenschild EH, Habets P, Jacobs HI, Mengelers R, Rozendaal N, Van Os J, et al. The effects of FreeSurfer version, workstation type, and Macintosh operating system version on anatomical volume and cortical thickness measurements. PloS one 2012;7(6):e38234.
7. Glatard T, Lewis LB, Ferreira da Silva R, Adalat R, Beck N, Lepage C, et al. Reproducibility of neuroimaging analyses across operating systems. Frontiers in neuroinformatics 2015;9:12.
8. Gummaraju J, Desikan T, Turner Y. Over 30% of official images in DockerHub contain high priority security vulnerabilities. In: Technical Report; 2015.
9. Shu R, Gu X, Enck W. A Study of Security Vulnerabilities on DockerHub. In: Proceedings of the Seventh ACM on Conference on Data and Application Security and Privacy CODASPY '17, New York, NY, USA; 2017. p. 269–280.
10. Zerouali A, Mens T, Robles G, Gonzalez-Barahona JM. On the relation between outdated Docker containers, severity vulnerabilities, and bugs. In: 2019 IEEE 26th International Conference on Software Analysis, Evolution and Reengineering (SANER) IEEE; 2019. p. 491–501.
11. Gorgolewski KJ, Alfaro-Almagro F, Auer T, Bellec P, Capotă M, Chakravarty MM, et al. BIDS apps: Improving ease of use, accessibility, and reproducibility of neuroimaging data analysis methods. PLoS computational biology 2017;13(3):e1005209.
12. Glatard T, Kiar G, Aumentado-Armstrong T, Beck N, Bellec P, Bernard R, et al. Boutiques: a flexible framework to integrate command-line applications in computing platforms. GigaScience 2018;7(5):giy016.
13. Rampin R, Chirigati F, Shasha D, Freire J, Steeves V. ReproZip: The Reproducibility Packer. Journal of Open Source Software 2016;1(8):107.
