# Supplementary material for: An analysis of security vulnerabilities in container images for scientific data analysis
Source: Gigascience. 2021 Jun 3;10(6):giab025. doi: 10.1093/gigascience/giab025 (PMC8173661; doi:10.1093/gigascience/giab025)
Supplement: giab025_Response_to_Reviewer_Comments_Original_Submission [file giab025_response_to_reviewer_comments_original_submission.pdf]

Author's Response To Reviewer Comments

Close

See letter and detailed answer included as attachment.

Close
