# Supplementary material for: An analysis of security vulnerabilities in container images for scientific data analysis
Source: Gigascience. 2021 Jun 3;10(6):giab025. doi: 10.1093/gigascience/giab025 (PMC8173661; doi:10.1093/gigascience/giab025)
Supplement: giab025_Response_to_Reviewer_Comments_Revision_1 [file giab025_response_to_reviewer_comments_revision_1.pdf]

# Author's Response To Reviewer Comments

Close

Dear Editor,

Thanks for the comments on our manuscript. In the revision attached, we have updated section "Availability of Data and Materials" and added a section on "Availability of Supporting Data" as per your comments. We remain available for any issue.

Best regards,
Tristan Glatard
(on behalf of the co-authors)

Close
