# Supplementary material for: An analysis of security vulnerabilities in container images for scientific data analysis
Source: Gigascience. 2021 Jun 3;10(6):giab025. doi: 10.1093/gigascience/giab025 (PMC8173661; doi:10.1093/gigascience/giab025)
Supplement: giab025_Reviewer_1_Report_Original_Submission — Chris Armit -- 11/11/2020 Reviewed [file giab025_reviewer_1_report_original_submission.pdf]

Reviewer Report

Title: An Analysis of Security Vulnerabilities in Container Images for Scientific Data Analysis

Version: Original Submission **Date:** 11/11/2020

Reviewer name: Chris Armit

Reviewer Comments to Author:

This very interesting Technical Note reports on security vulnerabilities in software container images used for neuroscience data analysis. The authors report on two software container images - Docker and Singularity - and four software container image scanners - Anchore, Vuls, Clair, and Stools. In their analysis, the authors highlight an average of 460 vulnerabilities per software container image. As a means of reducing the number of vulnerabilities, the authors explored the effects of package update and image minification. The authors highlight that updating container images is a major factor in reducing the number of vulnerabilities, and in support of this the authors report that no vulnerabilities were to be found in base Docker images ubuntu:20.04 and centos:7 after package update. The authors additionally report that removing unused packages by image minification is also effective. An important point that the authors demonstrate is that both image minification and package update techniques are complementary methods for reducing the number of security vulnerabilities.
This analysis enabled the authors to provide a set of image creation guidelines on how to deliver a more secure deployment of image containers on HPC clusters. These guidelines suggest minifying container images by using lightweight OS distributions, and applying regular security updates. These are very useful guidelines for the neuroscience data management community. The manuscript is well written, and the scripts used for container vulnerability analysis are publicly available on GitHub and have been ascribed an OSI-approved GPL3 license. The authors additionally provide a Jupyter notebook that can be used to regenerate the figures shown in the manuscript.
I recommend this Technical Note for publication in GigaScience.

Level of Interest

Please indicate how interesting you found the manuscript: Choose an item.

Quality of Written English

Please indicate the quality of language in the manuscript: Choose an item.

Declaration of Competing Interests

Please complete a declaration of competing interests, considering the following questions:

- Have you in the past five years received reimbursements, fees, funding, or salary from an organisation that may in any way gain or lose financially from the publication of this manuscript, either now or in the future?
- Do you hold any stocks or shares in an organisation that may in any way gain or lose financially from the publication of this manuscript, either now or in the future?
- Do you hold or are you currently applying for any patents relating to the content of the manuscript?
- Have you received reimbursements, fees, funding, or salary from an organization that holds or has applied for patents relating to the content of the manuscript?
- Do you have any other financial competing interests?
- Do you have any non-financial competing interests in relation to this paper?

If you can answer no to all of the above, write 'I declare that I have no competing interests' below. If your reply is yes to any, please give details below.

I declare that I have no competing interests.

I agree to the open peer review policy of the journal. I understand that my name will be included on my report to the authors and, if the manuscript is accepted for publication, my named report including any attachments I upload will be posted on the website along with the authors' responses. I agree for my report to be made available under an Open Access Creative Commons CC-BY license (http://creativecommons.org/licenses/by/4.0/). I understand that any comments which I do not wish to be included in my named report can be included as confidential comments to the editors, which will not be published.

Choose an item.

To further support our reviewers, we have joined with Publons, where you can gain additional credit to further highlight your hard work (see: https://publons.com/journal/530/gigascience). On publication of this paper, your review will be automatically added to Publons, you can then choose whether or not to claim your Publons credit. I understand this statement.

Yes Choose an item.
