# Supplementary material for: An analysis of security vulnerabilities in container images for scientific data analysis
Source: Gigascience. 2021 Jun 3;10(6):giab025. doi: 10.1093/gigascience/giab025 (PMC8173661; doi:10.1093/gigascience/giab025)
Supplement: giab025_Reviewer_2_Report_Original_Submission — Erik C. Johnson, Ph.D. -- 11/14/2020 Reviewed [file giab025_reviewer_2_report_original_submission.pdf]

Reviewer Report

Title: An Analysis of Security Vulnerabilities in Container Images for Scientific Data Analysis

Version: Original Submission **Date:** 11/14/2020

Reviewer name: Erik C. Johnson, Ph.D.

Reviewer Comments to Author:

This Technical Note manuscript makes several interesting investigations into the security of containers for neuroimaging processing, and the topic is suitable for Gigascience. The authors analyze a number of common, publicly available containerized images from widely used processing pipelines. The analysis of security vulnerabilities is extensive and results in a number of concrete pieces of advice and best practices for software developers in the neuroimaging space. There are also implications for other fields which commonly use containerized pipelines.

I have several suggestions for improvements of this manuscript, which I hope will improve the impact to the community.

While it is addressed at various points in the manuscript, the key issue within the neuroimaging community with regards to updating images and packages is the fundamental tension between reproducibility (by pinning the exact package versions and OS used) and security through using updated packages. The point needs to be made more clearly that security vulnerabilities need to be taken more seriously while providing more insight into the issues that can arise when updating packages. Some possible suggestions to address this:

1. In the introduction, you list several key questions. I think an additional question, or perhaps a modification to question two, is how much security vulnerabilities can be reduced without introducing errors or inconsistencies in processing data?
2. In the results, can you expand on the potential impact of the security vulnerabilities found? You highlight a few serious vulnerabilities, but have one sentence on the potential issues. Are there additional ramifications which would motivate the neuroimaging software developer to update? Perhaps jeopardizing data or user credentials to access online systems? This may allow you to be more specific in the last two paragraphs of the discussion as well in terms of actual security vulnerabilities found in these containers.
3. In the results, in the "Effect of image update" you mention "in spite of the associated reproducibility challenges"- did you run into any issues in these updates you can specifically report? This may provide insight into the process of upgrading neuroimaging containers.
4. In the results, in the "Effect of minification" you mention "It is a tedious operation, as it requires running an actual data analysis in the container image". I think this is also worth expanding on. Were there test examples, unit tests, or integration tests from BIDS or Boutiques used for this? Did the minification tools create any errors in these images? Was there any insight in which kinds of packages were removed? Were there commonalities in the packages which had security issues which could not be removed from minification?

Addressing these points would help the neuroscientist and neuroimaging software developer

understand the risks associated with outdated containers as well as the path to upgrade.
Some additional minor comments and questions
1. Were there significant differences between the kinds of errors found in docker and singularity images, or the potential severity of those errors given the improved user privilege control of singularity?
2. Does the docker engine or singularity engine version matter? Does the use of an orchestration system like docker compose matter? I am not sure but I am curious.
Even more minor comments:
1. Bigger text on Figure 1 would be helpful.

Level of Interest

Please indicate how interesting you found the manuscript: Choose an item.

Quality of Written English

Please indicate the quality of language in the manuscript: Choose an item.

Declaration of Competing Interests

Please complete a declaration of competing interests, considering the following questions:

- Have you in the past five years received reimbursements, fees, funding, or salary from an organisation that may in any way gain or lose financially from the publication of this manuscript, either now or in the future?
- Do you hold any stocks or shares in an organisation that may in any way gain or lose financially from the publication of this manuscript, either now or in the future?
- Do you hold or are you currently applying for any patents relating to the content of the manuscript?
- Have you received reimbursements, fees, funding, or salary from an organization that holds or has applied for patents relating to the content of the manuscript?
- Do you have any other financial competing interests?
- Do you have any non-financial competing interests in relation to this paper?

If you can answer no to all of the above, write 'I declare that I have no competing interests' below. If your reply is yes to any, please give details below.

I declare that I have no competing interests

I agree to the open peer review policy of the journal. I understand that my name will be included on my report to the authors and, if the manuscript is accepted for publication, my named report including any attachments I upload will be posted on the website along with the authors' responses. I agree for my

report to be made available under an Open Access Creative Commons CC-BY license (http://creativecommons.org/licenses/by/4.0/). I understand that any comments which I do not wish to be included in my named report can be included as confidential comments to the editors, which will not be published.

Choose an item.

To further support our reviewers, we have joined with Publons, where you can gain additional credit to further highlight your hard work (see: https://publons.com/journal/530/gigascience). On publication of this paper, your review will be automatically added to Publons, you can then choose whether or not to claim your Publons credit. I understand this statement.

Yes Choose an item.
