# Supplementary material for: An analysis of security vulnerabilities in container images for scientific data analysis
Source: Gigascience. 2021 Jun 3;10(6):giab025. doi: 10.1093/gigascience/giab025 (PMC8173661; doi:10.1093/gigascience/giab025)
Supplement: giab025_Reviewer_3_Report_Original_Submission — Yaroslav Halchenko -- 11/30/2020 Reviewed [file giab025_reviewer_3_report_original_submission.pdf]

Reviewer Report

Title: An Analysis of Security Vulnerabilities in Container Images for Scientific Data Analysis

Version: Original Submission **Date:** 11/30/2020

Reviewer name: Yaroslav Halchenko

Reviewer Comments to Author:

* Review summary
This paper presents a little study of selected set of popular containerized applications (BIDS-Apps, Boutiques) regarding the number of known vulnerabilities found to be present in the contained within containers software components. Two approaches to reduce number of known/detected vulnerabilities were approached: container updates and/or minimization. Overall it is a nice and informative paper. I only have once notable concern: recommendation "ii. Use lightweight base images such as Alpine Linux" . As described in greater detail below, I think at large it would only potentially only "hide" the problem away by making vulnerabilities undetected (but not "not present").
* Abstract
** ", [especially] on high-performance computing clusters (HPC)".
Since the statement is IMHO applicable in general, not only to HPC.
Similar statement in Introduction opening.
I could even argue that taking "possession" over a local desktop could provide an attack vector enabling intruder to access multiple HPC systems a user might have access to.
So I would not "limit" to HPC alone, but rather add a statement that such vulnerabilities might affect HPC deployments at a larger scale, while circumventing/obliterating security measures system administrators put in place.
* Introduction
** "and [often can] control the memory, CPU, network and file-system resources"
Since AFIAK Singularity by default would not bother to control memory/CPU/network or even file resources. That was the point behind it: to be (unlike Docker) very transparent to the process and as close to the "chroot" lightweight as possible.
* "for Docker and appc container"
may be that originally used version promoted support for appc, but as appc specification was stopped to be developed in favor of OCI, Clair now states support for OCI (not appc). So it might be better

to say "Docker and OCI container images"
* "give scanning results" -> "list scanning results"
 minor, and I do not like "list" either but have not come up with a
 better alternative
* "In comparison, no vulnerabilities were found in base Docker images ubuntu:20.04 and centos:7 after package update. "
 That it IMHO would be inappropriate comparison to make: non-updated
 applications images to updated stock images. Why not to also
 provide a number of vulnerabilities in base images **BEFORE** the
 update? That would also deliver the point that even base images
 could have vulnerabilities.
 If no longer possible to do easily (no access to the original base
 images), just remove "In comparison,"?
* Figure 1 -- I cannot tell between "critical" and Ubuntu -- both are vivid red
 Since it has a (n) anyways, why just to make it that solid color
 which is for High ATM and adjust all the rest accordingly? would
 make figure more consistent IMHO
* "six of them could not be updated due to various issues with the package manager,"
 too vague. Most likely it was not an "issue" per se but a. base
 distribution is EOLed and APT lines had to be adjusted (was not
 done), or key expired (also could be worked around). I would have
 advised to rephrase with a bit more clearer statement on why they
 were not updated - as "update" is promoted as an effective way to
 address vulnerabilities, inability (or difficulty) to update is an
 important factor!
* Is minimization "useful" to address security issues?
 Please state and support your opinion, since IMHO minimization is of
 no direct effect since vulnerable minimized-away software packages
 (even though shipped within container) are not involved in the
 computation anyways. Describe how/when they could potentially be
 harmful (e.g. a user unintentionally triggers those packages
 execution, which should be unlikely if container user through its
 computational entry point).
 The coin could also be flipped: only a port of vulnerabilities would
 in the containers could be relevant to the computational workflow.
 Judging from Fig 1B it actually could be 25-100% (I suspect g was
 minified by its developers)
* Discussion
** i. Introduce software dependencies cautiously
  agree.
  you could provide immediate hints such as `--no-install-recommends`
  for `apt install` invocations.

For this and the rest of the recommendations, I think readers would
greatly benefit from addition of more specific references and
examples, e.g. for "vi. Run image scanners during continuous
integration" -- is there a project/container you could refer to as
an example?

** ii. Use lightweight base images such as Alpine Linux.
disagree, since it could also fire back.
I hypothesize: The fact that you found less vulnerabilities on
alpine-based images could be due to the fact that dependencies were
manually built/installed (or some other distribution like conda was
used) and thus such "custom" installations simply were not scanned
by vulnerability scanners. "update" of such containers becomes
infeasible.
As a result you just end up just amplifying the problem:
vulnerabilities cannot be detected (but exist), updates are not
possible. Yes, such images would be "smaller", which will be good,
but you are hiding the elephant with such suggestion.
Similarly a statement in the conclusions should be adjusted, which
ATM just recommends "using lightweight OS distributions".

** iii. Use OS releases with long-term support.
You should note that "base OS" LTS or not support relates to only
packages provided through the distribution. It provides no magical
means for vulnerabilities fixup for scientific software installed
manually or from additional repositories (like NeuroDebian,
NeuroFedora, conda-forge, etc). BUT if scientific software components
are built "properly", dynamically linking against distribution
provided libraries, then updates of the base distribution would
automagically address vulnerabilities within scientific components
(which would not happen if they are either built statically embedding
all the libraries, or just bundling them for distribution -- e.g. like
standalone distributions of FSL, FreeSurfer etc would do).
So, by itself, stable base is not a guarantee that container would
be "safer" after update. Scientific components should ideally be
integrated within the distribution itself. The follow up
"iv. Install packages, not files" is a good one, but IMHO text here
should be a bit more explicit on aforementioned aspect.
It could also "link" into that aspect of "could not be updated due
to various issues with the package manager," -- how many of those
were not based on LTS of some kind?

*** "and Debian stable releases are maintained for 3 years"
There is Debian LTS support, after "stable release support", which
is provided for at least 5 years: https://wiki.debian.org/LTS

** "v. Minify container images. "
and explicitly 'link' to i. on cautious introduction of dependencies? might even better be reordered so those two advises come close.

Level of Interest

Please indicate how interesting you found the manuscript: Choose an item.

Quality of Written English

Please indicate the quality of language in the manuscript: Choose an item.

Declaration of Competing Interests

Please complete a declaration of competing interests, considering the following questions:

- Have you in the past five years received reimbursements, fees, funding, or salary from an organisation that may in any way gain or lose financially from the publication of this manuscript, either now or in the future?
- Do you hold any stocks or shares in an organisation that may in any way gain or lose financially from the publication of this manuscript, either now or in the future?
- Do you hold or are you currently applying for any patents relating to the content of the manuscript?
- Have you received reimbursements, fees, funding, or salary from an organization that holds or has applied for patents relating to the content of the manuscript?
- Do you have any other financial competing interests?
- Do you have any non-financial competing interests in relation to this paper?

If you can answer no to all of the above, write 'I declare that I have no competing interests' below. If your reply is yes to any, please give details below.

I declare that I have no competing interests

I agree to the open peer review policy of the journal. I understand that my name will be included on my report to the authors and, if the manuscript is accepted for publication, my named report including any attachments I upload will be posted on the website along with the authors' responses. I agree for my report to be made available under an Open Access Creative Commons CC-BY license (http://creativecommons.org/licenses/by/4.0/). I understand that any comments which I do not wish to be included in my named report can be included as confidential comments to the editors, which will not be published.

Choose an item.

To further support our reviewers, we have joined with Publons, where you can gain additional credit to further highlight your hard work (see: https://publons.com/journal/530/gigascience). On publication of this paper, your review will be automatically added to Publons, you can then choose whether or not to claim your Publons credit. I understand this statement.

Yes Choose an item.
